# Ferroptosis: molecular mechanisms and health implications

**DOI:** 10.1038/s41422-020-00441-1

**Published:** 2020-12-02

**Authors:** Daolin Tang, Xin Chen, Rui Kang, Guido Kroemer

**Affiliations:** 1 Guangzhou Municipal and Guangdong Provincial Key Laboratory of Protein Modification and Degradation; The Third Affiliated Hospital; Guangzhou Medical University, Guangzhou, Guangdong 511436 China; 2 Department of Surgery, UT Southwestern Medical Center, Dallas, TX 75390 USA; 3 Equipe Labellisée par la Ligue Contre le Cancer, Université de Paris, Sorbonne Université, INSERM U1138, Centre de Recherche des Cordeliers, Paris, France; 4 Metabolomics and Cell Biology Platforms, Gustave Roussy Cancer Campus, Villejuif, 94800 France; 5 Pôle de Biologie, Hôpital Européen Georges Pompidou, AP-HP, Paris, 75015 France; 6 Suzhou Institute for Systems Biology, Chinese Academy of Sciences, Suzhou, Jiangsu China; 7 Department of Women’s and Children’s Health, Karolinska University Hospital, Stockholm, 17176 Sweden

**Keywords:** Cell biology, Molecular biology

## Abstract

Cell death can be executed through different subroutines. Since the description of ferroptosis as an iron-dependent form of non-apoptotic cell death in 2012, there has been mounting interest in the process and function of ferroptosis. Ferroptosis can occur through two major pathways, the extrinsic or transporter-dependent pathway and the intrinsic or enzyme-regulated pathway. Ferroptosis is caused by a redox imbalance between the production of oxidants and antioxidants, which is driven by the abnormal expression and activity of multiple redox-active enzymes that produce or detoxify free radicals and lipid oxidation products. Accordingly, ferroptosis is precisely regulated at multiple levels, including epigenetic, transcriptional, posttranscriptional and posttranslational layers. The transcription factor NFE2L2 plays a central role in upregulating anti-ferroptotic defense, whereas selective autophagy may promote ferroptotic death. Here, we review current knowledge on the integrated molecular machinery of ferroptosis and describe how dysregulated ferroptosis is involved in cancer, neurodegeneration, tissue injury, inflammation, and infection.

## Introduction

**Fig. 1:**
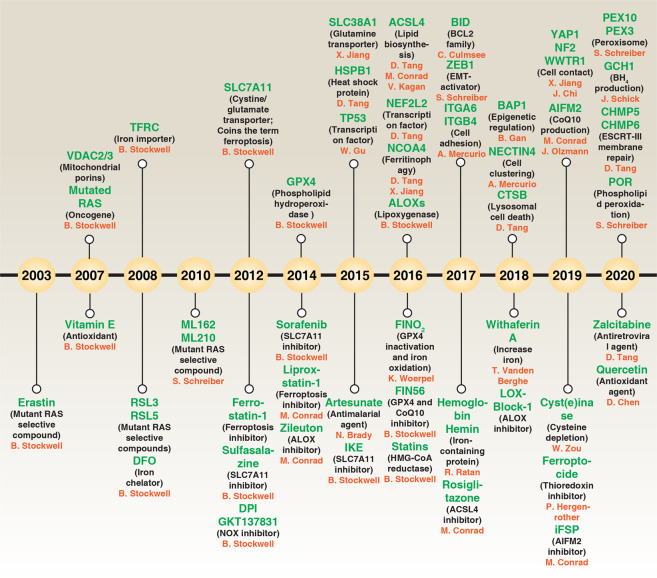
Timeline of ferroptosis research. A brief history of molecular and pharmacological modulators of ferroptosis.

Over the past decades, several forms of cell death were identified and classified as accidental or regulated cell deaths.^1^ Unlike accidental cell death (an uncontrolled passive process), regulated (active) cell death can be mediated through a series of molecular mechanisms and signaling pathways.^2^ The best-studied form of regulated cell death is apoptosis, which is mainly triggered by the activation of proteases from the caspase family. Non-apoptotic cell death has recently attracted widespread attention in tumor therapy because resistance to apoptosis is a hallmark of cancer. One such non-apoptotic modality of cell death, ferroptosis, is defined as an iron-dependent regulated necrosis that is caused by massive lipid peroxidation-mediated membrane damage.^3^ The toxicity of iron and lipid peroxidation were reported in the 1900s and 1950s, respectively.^4,5^ As an evolutionary conservative program, ferroptosis plays a vital role in the development and disease of various organisms, including the plant and animal kingdoms.^6,7^ Although the term “ferroptosis” was coined in 2012 after screenings for small-molecule compounds capable of inhibiting the growth of RAS-mutant cancer cells^8^ (Fig. 1), the initial theoretical idea of ferroptosis may have developed from nutrient (in particular cysteine) depletion-induced cancer cell death^9^ and “oxytosis”, the death of neurons succumbing to the excitotoxin glutamate and simultaneous inhibition of the amino acid antiporter solute carrier family 7 member 11 (SLC7A11/xCT/system xc^−^).^10–12^ Recently, multiple pharmacological or natural compounds (Supplementary information, Table S1) and cell-intrinsic proteins (Supplementary information, Table S2) have been reported to regulate the process and function of ferroptotic cell death. Here, we will summarize the emerging regulatory network that affects ferroptosis, as well as its potential pathogenic role in diseases, while emphasizing unresolved questions in this area.

## Hallmarks of ferroptosis

### Morphological features

Although an initial study indicated that ferroptosis is morphologically, biochemically and genetically distinct from apoptosis, necrosis and autophagy,^8^ most investigators concur that cells undergoing ferroptosis usually show necrosis-like morphological changes.^13^ These features include a loss of plasma membrane integrity, cytoplasmic swelling (oncosis), swelling of cytoplasmic organelles and moderate chromatin condensation. In some cases, ferroptosis is also accompanied by detachment and rounding up of cells, as well as by increased autophagosomes.^8,14–16^ Of note, ferroptosis occurring in one cell can reportedly spread to adjacent cells in a fast-propagating wave.^17,18^ At the ultrastructural level, ferroptotic cells usually exhibit mitochondrial abnormalities, such as condensation or swelling, increased membrane density, reduced or absent crista, as well as rupture of the outer membrane.^8,15,16^ Despite these significant changes in mitochondrial morphology, the role of these organelles in ferroptosis remains controversial. Mitochondria are the center of metabolism and an important source of reactive oxygen species (ROS) in most mammalian cells. At odds with an early study indicating that mitochondria-mediated ROS production is not necessary for ferroptosis,^8^ more recent evidence indicates that mitochondrion-mediated ROS production, DNA stress, and metabolic reprogramming are required for lipid peroxidation and ferroptosis induction.^19–21^

### Biochemical features

**Fig. 2:**
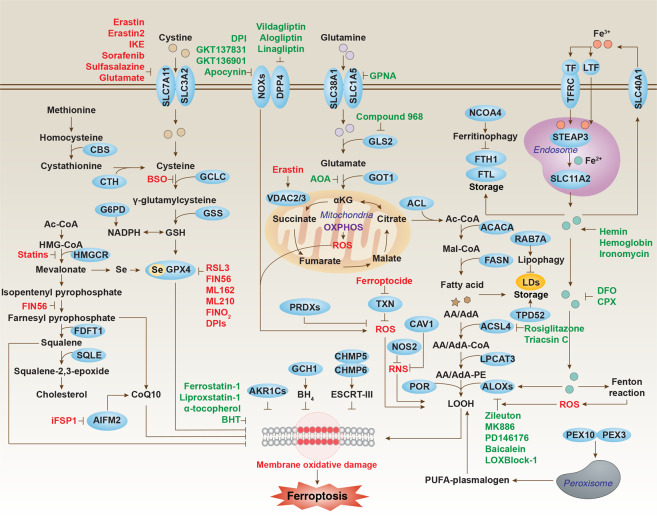
Core molecular machinery and signaling regulation of ferroptosis. Ferroptosis can occur through two major pathways, the extrinsic or transporter-dependent pathway (e.g., decreased cysteine or glutamine uptake and increased iron uptake), and the intrinsic or enzyme-regulated pathway (e.g., the inhibition of GPX4).

Ferroptosis is a ROS-dependent form of cell death associated with two main biochemical characteristics, namely iron accumulation and lipid peroxidation (Fig. 2).

#### Iron accumulation

The classical ferroptosis activators erastin or RSL3 inhibit the antioxidant system as they increase intracellular iron accumulation.^8^ Iron may directly generate excessive ROS through the Fenton reaction, thereby increasing oxidative damage.^8^ In addition, iron may increase the activity of lipoxygenase (ALOX) or EGLN prolyl hydroxylases (also called PHD), which are enzymes responsible for lipid peroxidation and oxygen homeostasis. The dynamics between systemic and local cellular iron regulation affects the sensitivity of ferroptosis.^22^ Targeting genes related to iron overload, or the use of iron-chelating agents, effectively inhibits ferroptotic cell death (discussed later). It is unclear why only iron, but not other metals (such as zinc) that also cause ROS production through a Fenton reaction,^23^ has the ability to induce ferroptosis.^8^ One possibility is that iron overload activates specific downstream effectors that contribute to the execution of ferroptosis after the production of lipid ROS.

#### Lipid peroxidation

Lipid peroxidation is a free radical-driven reaction, which mainly affects unsaturated fatty acids in the cell membrane. The products of lipid peroxidation include the initial lipid hydroperoxides (LOOHs) and subsequent reactive aldehydes (e.g., malondialdehyde (MDA) and 4-hydroxynonenal (4HNE)), which increase during ferroptosis. There are three types of fatty acids, namely, saturated fatty acids (no double bond), monounsaturated fatty acids (MUFAs, 1 double bond) and polyunsaturated fatty acids (PUFAs, >1 double bond). Although various cell membrane lipids (e.g., phosphatidylcholine, phosphatidylethanolamine (PE) and cardiolipin) may be oxidized, peroxidation of PUFAs in phospholipids by ALOXs appears to be particularly important for ferroptosis.^24,25^ Although mitochondria undergo strong changes during ferroptosis, cardiolipin peroxidation is not found in ferroptosis.^26^

### Genetic features

The overexpression of a few genes/proteins has been considered a biomarker of ferroptosis, as exemplified by prostaglandin-endoperoxide synthase 2 (PTGS2/COX2), the key enzyme in prostaglandin biosynthesis.^27^ However, PTGS2 does not use prostaglandins as a substrate for lipid peroxidation in ferroptosis. Acyl-CoA synthetase long-chain family member 4 (ACSL4), an enzyme involved in fatty acid metabolism, is considered as a specific biomarker and driver of ferroptosis because the upregulation of ACSL4 enhances the PUFA content in phospholipids, which are susceptible to oxidation reactions leading to ferroptosis.^24,28,29^ Nonetheless, ACSL4 is not required for ferroptosis in all cases, meaning ACSL4-depleted cells can undergo ferroptosis in specific circumstances.^30^ The activation of genes responsible for antioxidant defense (e.g., the glutathione (GSH) system,^8^ coenzyme Q10 (CoQ10) system,^31,32^ and the nuclear factor, erythroid 2-like 2 (NFE2L2, also known as NRF2) transcription pathway^33^) and membrane repair (e.g., the endosomal sorting complexes required for transport (ESCRT)-III pathway^34^) limit membrane damage during ferroptosis (Fig. 2). Thus, depending on the balance of injury and anti-injury responses, cells “decide” to live or to die in response to ferroptotic stimuli.

### Immune features

**Fig. 3:**
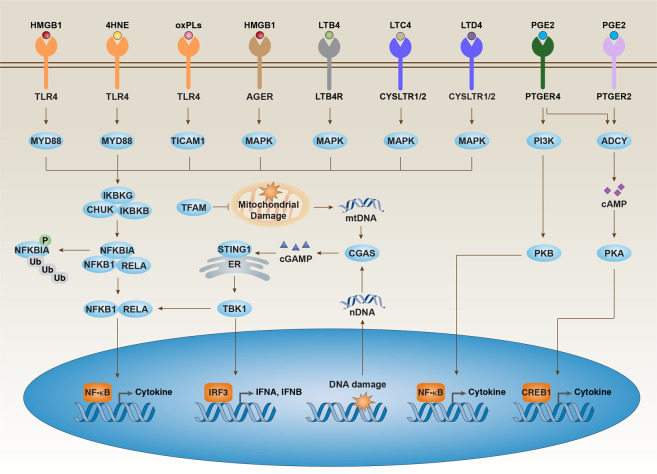
Immune features of ferroptosis. Ferroptotic cell death can lead to different immune and inflammatory reactions through the release and activation of damage-associated molecular patterns (e.g., HMGB1 and DNA) or lipid oxidation products (e.g., 4HNE, oxPLs, LTB4, LTC4, LTD4, and PGE2) in immune cells (e.g., macrophages, monocytes, and neutrophils) via different intracellular signal transduction pathways.

The immunological consequences of ferroptosis involve two aspects. First, ferroptosis can lead to the death of leukocyte subsets and the corresponding loss of immune function. For example, lipid peroxidation-induced ferroptosis in T cells promotes viral or parasitic infections.^35^ Second, and more importantly, when ferroptosis affects non-leukocytic cells, it determines how dying cells or the resulting corpses are handled by the immune system. Different types of cell death can lead to different immune and inflammatory reactions through the release and activation of different damage-associated molecular pattern (DAMP) signals. In general, ferroptosis is a form of inflammatory cell death associated with the release of DAMPs (e.g., high mobility group box 1 (HMGB1) and DNA) or lipid oxidation products (e.g., 4HNE, oxPLs, LTB4, LTC4, LTD4 and PGE2) during tissue injury or tumor therapy (Fig. 3). For example, in aging and chronic diseases, the lipid peroxidation product 4HNE is a pro-inflammatory mediator that activates the nuclear factor-κB (NF-κB) pathway.^36^ HMGB1, a prototypical DAMP involved in various types of cell death,^37^ is released by ferroptotic cells and then triggers an inflammatory response in peripheral macrophages through the activation of advanced glycosylation end-product-specific receptor (AGER/RAGE), a pattern-recognition receptor activating the NF-κB pathway in innate immunity.^38^ Targeting lipid metabolism-related DAMP signaling might be a promising strategy for the treatment of inflammatory diseases related to ferroptotic damage.

## Regulation of ferroptosis

### Iron metabolism

Iron has two oxidation states: ferrous (Fe^2+^) or ferric (Fe^3+^). The iron redox cycle may affect the sensitivity of cells to ferroptosis (Fig. 2). Non-heme iron in food is mainly Fe^3+^, which is insoluble, and needs to be reduced to Fe^2+^ for absorption. Hemin and ferric ammonium citrate, but not biliverdin and bilirubin, promote erastin or FINO_2_-induced ferroptotic cell death.^39^ Fe^3+^ binds to transferrin (TF) in the serum and then is recognized by TFRC in the cell membrane. Similar to TF,^40^ lactotransferrin (LTF) also functions as a positive regulator of ferroptosis by increasing iron intake.^41^ Actin filaments are one of the main components of the cytoskeleton and participate in TFRC-mediated iron absorption. Protein kinase C (PKC)-mediated phosphorylation of heat shock protein family B (small) member 1 (HSPB1/HSP25/HSP27) limits the cytoskeleton-mediated iron uptake and therefore limits ferroptotic cancer cell death.^42^

After absorption by TFRC, the STEAP3 metalloreductase in the endosome reduces Fe^3+^ to Fe^2+^, and then releases Fe^2+^ from the endosome into the cytosol through solute carrier family 11 member 2 (SLC11A2/DMT1). Fe^2+^ is important for metabolic and biochemical processes, such as oxygen transport, energy metabolism and iron-sulfur protein production in mitochondria. As a cofactor, iron regulates the activity of iron-requiring enzymes by forming redox-active (loosely-bound) and redox-silent complexes, thereby playing a complex role in oxidative stress.^43^ The inhibition of iron–sulfur cluster biosynthesis by the depletion of NFS1 cysteine desulfurase promotes erastin-induced ferroptosis.^44^ The overexpression of the iron–sulfur cluster assembly enzyme (ISCU) attenuates dihydroartemisinin (DHAN)-induced ferroptosis in leukemia cells.^45^ These findings indicate that decreasing iron utilization may increase the sensitivity to ferroptosis.^44^ The iron–sulfur cluster proteins CDGSH iron domain 1 (CISD1/mitoNEET) and CISD2 inhibit ferroptosis by reducing mitochondrial iron uptake.^46,47^ Abnormal increases in Fe^2+^ in lysosomes and endoplasmic reticulum (ER) may promote ferroptosis, indicating that various intracellular iron pools promote ferroptosis.

**Fig. 4:**
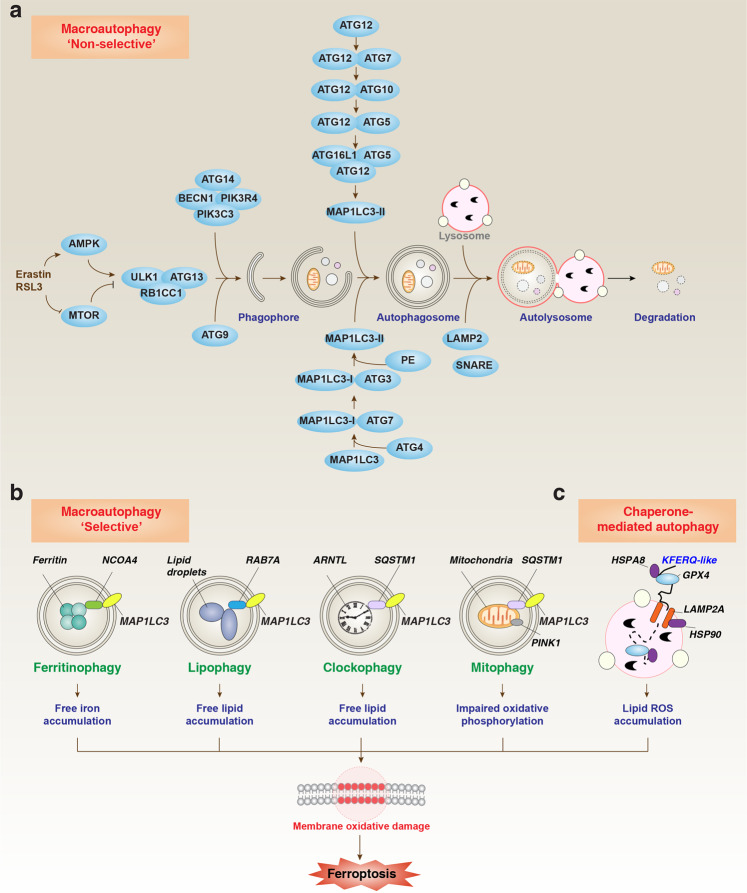
Role of autophagy in ferroptosis. **a** The mechanism of non-selective macroautophagy/autophagy induced by ferroptosis activator (e.g., erastin and RSL3). **b**, **c** Certain selective types of autophagy (e.g., ferritinophagy, lipophagy, clockophagy and mitophagy) (**b**) and chaperone-mediated autophagy (**c**) promote oxidative damage-dependent ferroptosis through the degradation of ferroptosis repressors (e.g., ferritin, ARNTL/BMAL1, lipid droplets and GPX4).

The iron-storage protein ferritin includes ferritin light chain (FTL) and ferritin heavy chain 1 (FTH1), which can be degraded by lysosomes to increase free iron levels. Inhibiting nuclear receptor coactivator 4 (NCOA4)-mediated ferritinophagy, a type of selective autophagy for the degradation of ferritin by lysosomes, increases iron storage and limits ferroptosis in cancer cells^48,49^ (Fig. 4a, b). Moreover, the overexpression of ferritin mitochondrial (FTMT), an iron-storage protein in mitochondria, inhibits erastin-induced ferroptosis in neuroblastoma cells,^50^ indicating a wide anti-ferroptotic role for iron-storage proteins. Poly(RC)-binding proteins (PCBPs) act as iron chaperones, delivering iron to the corresponding protein client. PCBP1 delivers Fe^2+^ to ferritin, thereby limiting ferroptosis in hepatocytes.^51^

Finally, the iron-efflux protein solute carrier family 40 member 1 (SLC40A1/ferroportin1/FPN) extrudes iron into the extracellular space, as Fe^2+^ is reoxidized to Fe^3+^ by ferroxidases (e.g., ceruloplasmin (CP) or hephaestin (HEPH)). The overexpression of SLC40A1 inhibits, whereas the knockdown of SLC40A1 increases, siramesine and lapatinib-induced ferroptosis through the modulation of iron efflux in breast cancer cells.^52^ Alternatively, prominin 2 (PROM2), a member of the prominin family of pentaspan membrane glycoproteins, causes ferroptosis resistance by stimulating exosome-dependent iron export through the formation of ferritin-containing multivesicular bodies in epithelial and breast carcinoma cells.^53^ Therefore, blocking the iron release pathway on cell membranes increases the susceptibility to ferroptosis.

**Fig. 5:**
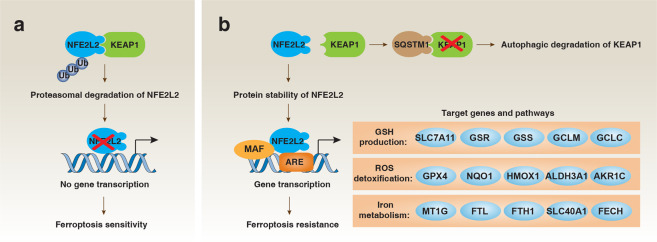
NFE2L2 in ferroptosis. **a** Under normal conditions, a low level of NFE2L2 is primarily maintained by KEAP1-mediated proteasomal degradation. **b** Following ferroptosis stress, the NFE2L2 protein is stabilized and initiates a multi-step activation pathway, including nuclear translocation, heterodimerization with its partner MAF protein, recruitment of transcription coactivators and subsequent binding to the antioxidant response element (ARE) of the target gene promoter. SQSTM1 can stabilize NFE2L2 by inactivating KEAP1 through autophagic degradation.

Genes related to iron metabolism are generally upregulated during ferroptosis. The silencing of iron-responsive element-binding protein 2 (IREB2), a major regulator of iron metabolism, affects TFRC, ISCU, FTH1 and FTL expression during ferroptosis.^8^ NFE2L2 is a transcription factor that regulates heme and iron metabolism through the transcriptional upregulation of multiple genes, including heme oxygenase 1 (*HMOX1*/*HO1*) (Fig. 5a, b). However, HMOX1 plays a dual role in ferroptosis.^33,39^ This dual mechanism of protection versus cytotoxicity of HMOX1 was already proposed in 1999.^54^ Ferroptosis caused by excessive HMOX1 activity is called non-canonical ferroptosis.^55^ ATM serine/threonine kinase, a DNA-damage response protein, promotes ferroptosis through inhibiting nuclear translocation of metal regulatory transcription factor 1 (MTF1), a transcription factor that induces the expression of *SLC40A1*, *FTH1*, and *FTL* to limit iron toxicity.^56^ Together, these findings provide evidence that multiple factors control iron metabolism in ferroptosis.^22^

### Lipid metabolism

#### Lipid synthesis

The peroxidation of PUFAs at the bis-allylic position is an important step in promoting ferroptosis.^57^ Increased synthesis of PUFA promotes subsequent lipid peroxidation under oxidative stress conditions. PUFA may possess unsaturated bonds in the omega-6 (e.g., linoleic acid (LA; 18:2), gamma-linolenic acid (GLA; 18:3), dihomo-gamma-linolenic acid (DGLA; 20:3), arachidonic acid (AA; 20:4) and adrenic acid (AdA; 22:4)) or omega-3 positions (e.g., alpha-linolenic acid (ALA; 18:3), eicosapentaenoic acid (EPA; 20:5) and docosahexaenoic acid (DHA; 22:6)) and have amphiphilic properties to maintain the fluidity of the cell membrane. AA and AdA (hereafter called AA/AdA) are the main substrates of lipid peroxidation in ferroptosis.^24^ Phospholipase A2 (PLA2) cleaves PUFAs into free PUFAs and lysophospholipids. The analysis of RSL3- or ML162-resistant KBM7 cells (a haploid chronic myeloid leukemia cell line) indicates two genes significantly enriched for gene trap insertions, namely *ACSL4* and lysophosphatidylcholine acyltransferase 3 (*LPCAT3*).^58^ Indeed, the production of AA/AdA derivatives for ferroptosis requires ACSL4 and LPCAT3.^24,28,29^ ACSL4 first catalyzes the biochemical reaction of free AA/AdA to CoA to form AA/AdA-CoA derivatives and promotes their esterification into phospholipids, while LPCAT3 then catalyzes the biosynthesis of AA/AdA-CoA and membrane PE to form AA/AdA–PE (Fig. 2). Consequently, the inhibition of ACSL4 or LPCAT3 diminishes ferroptosis in various conditions. Omega-6, but not omega-3, PUFAs restore ferroptosis sensitivity in ACSL4-deficient cells.^29^ Dietary supplementation with mixed omega-6 and omega-3 PUFAs can favor the development of ferroptosis-related inflammatory bowel disease in mice.^59^ Thus, both omega-6 and omega-3 PUFAs seem to play a role in ferroptosis. Recently, the synthesis of plasmalogen stimulated by peroxisomal biogenesis factor 10 (PEX10) and PEX3 has been proposed as another source of PUFAs, suggesting that peroxisomes may contribute to lethal ferroptosis-associated lipid peroxidation.^60^

The activity of PUFAs in ferroptosis is competitively affected by MUFAs, meaning that exogenous MUFAs (e.g., oleic acid (C18:1) and palmitoleic acid (POA; 16:1 cis-7)) cause ferroptosis resistance.^57,61,62^ MUFAs do not have bis-allylic positions, hence are not readily peroxidized. This MUFA-induced ferroptosis resistance relies on ACSL3 or stearoyl-CoA desaturase (SCD/SCD1, an enzyme involved in fatty acid biosynthesis, primarily the synthesis of oleic acid), instead of ACSL4.^61,62^ However, the MUFA oleic acid can induce ferroptosis in mice with acute lung injury,^63^ arguing that MUFA-mediated ferroptosis modulation is context-dependent. Deuterated PUFAs (D-PUFAs) at bis-allylic position reduce oxidative stress to protect against erastin- or RSL3-induced ferroptosis,^57^ indicating that the activity of PUFAs in ferroptosis is also regulated by their structure.

#### Lipid peroxidation

**Fig. 6:**
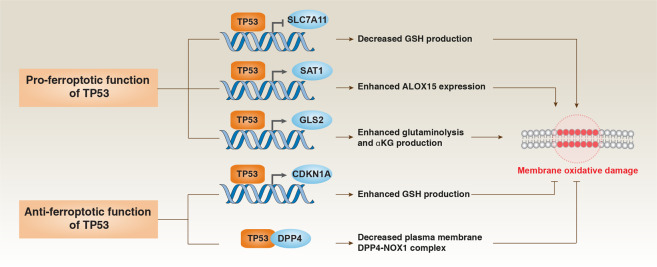
TP53 in ferroptosis. TP53 plays a context-dependent role in the regulation of membrane oxidative damage in ferroptosis. On the one hand, TP53 can enhance ferroptosis by inhibiting SLC7A11 expression or promoting SAT1 and GLS2 expression. On the other hand, TP53 can inhibit ferroptosis by inhibiting DPP4 activity or inducing CDKN1A expression.

The mammalian ALOX family, consisting of six members (ALOXE3, ALOX5, ALOX12, ALOX12B, ALOX15 and ALOX15B), plays a tissue- or cell-dependent role in mediating PUFA peroxidation to produce AA/AdA-PE-OOHs, thus causing ferroptosis. For example, spermidine/spermine N1-acetyltransferase 1 (*SAT1*), a tumor protein p53 (TP53) target gene, mediates the expression of ALOX15 (but not ALOX5 and ALOX12) and is involved in TP53-mediated ferroptosis in H1299 cells.^64^ In contrast, ALOX12, but not other ALOX members, is required for ferroptosis caused by TP53-mediated downregulation of SLC7A11 in H1299 cells.^30^ Therefore, different TP53 pathways utilize different ALOXs to induce ferroptosis (Fig. 6).

In vivo, ALOX12/15 is not required for glutathione peroxidase 4 (GPX4) depletion-induced ferroptotic damage in the kidney^16^ and T cells,^35^ whereas ALOX15 is essential for erastin- or RSL3-induced ferroptotic cell death through binding to the partner phosphatidylethanolamine-binding protein 1 (PEBP1, a multifunctional adapter protein) that allows ALOX15 to recognize stearoyl-AA–PE to generate lipid peroxides.^25^ These lipid peroxides may undergo secondary chemical reactions to produce electrophilic oxidative truncations that are prone to interact with nucleophilic sites in proteins. This lipid–protein interaction may further form a network that controls ferroptosis sensitivity.

ALOXs may not be the only regulators of lipid peroxidation in ferroptosis. Indeed, cytochrome P450 oxidoreductase (POR) combines with two cofactors (flavin mononucleotide (FMN) and flavin adenine dinucleotide (FAD)) to directly supply electrons to the P450 enzyme from nicotinamide adenine dinucleotide phosphate (NADPH, an essential electron donor in all organisms), thereby promoting PUFA peroxidation in cancer cells in an ALOX-independent manner^65^ (Fig. 2). It remains unknown whether other oxygenases, such as cyclooxygenases and peroxygenases, play a similar role in lipid peroxidation. PTGS2/COX2 does not directly oxidize phospholipids, but can oxidize lysophospholipids. PTGS2 is usually considered as a biomarker, but not as a driver, of ferroptosis.^27^ However, PTGS2 may mediate ferroptosis in neural cells after traumatic injury in the brain.^66^

#### Lipid storage and degradation

Intracellular lipids are stored in ER-derived lipid droplets and metabolized by cytoplasmic neutral hydrolases to provide lipids for cellular metabolism. In response to oxidative damage, the formation of lipid droplets can prevent cell death by separating PUFA from membrane phospholipids. Although lipid droplets are not essential for exogenous MUFA-mediated ferroptosis inhibition,^61^ the selective degradation of lipid droplets by RAB7A-related autophagy (namely lipophagy) increases the production of free fatty acids and promotes lipid peroxidation and subsequent ferroptosis^67^ (Fig. 4b). In contrast, tumor protein D52 (TPD52)-dependent lipid storage inhibits RSL3-induced ferroptosis in HCC cells^67^ (Fig. 2). Perilipin 2 (PLIN2), a member of the lipid droplet protein family, also inhibits ferroptosis in gastric cancer cells.^68^ Squalene is a lipophilic metabolite that can accumulate in cell membranes and lipid droplets. The loss of squalene epoxidase (SQLE) causes squalene accumulation, leading to ferroptosis resistance in anaplastic lymphoma kinase (ALK)-positive anaplastic large-cell lymphoma cells.^69^ Thus, increased lipid storage generally limits, whereas increased lipid degradation promotes ferroptosis.

#### Lipid transport and β-oxidation

Sterol carrier protein 2 (SCP2)-mediated trafficking of peroxidized lipids to mitochondria promotes GPX4 depletion-induced ferroptosis, supporting a role for mitochondria in ferroptosis.^16^ During β-oxidation, fatty acid molecules are broken down by removing two carbon units from the carboxyl end of a fatty acid molecule to produce acetyl-CoA. Interestingly, etomoxir, a potential inhibitor of β-oxidation of PUFA, does not suppress but rather stimulates RSL3-induced cell death,^24^ indicating it may have off-target effects. Acetyl-CoA carboxylase alpha (ACACA/ACC1), a central enzyme involved in fatty acid β-oxidation and fatty acid biosynthesis, plays a context-dependent role in promoting ferroptosis (Fig. 2). The silencing of ACACA in HT1080 cells results in resistance to FIN56, but not RSL3.^58,70^ However, the inhibition of ACACA blocks both erastin- and RSL3-induced ferroptosis in MEFs.^21^ A better understanding of fatty acid turnover in cells may help to deconvolute the intricate regulation of ferroptosis by different lipids.

## Oxidant system

Free radicals, including ROS and reactive nitrogen species (RNS), are oxidants produced by redox reactions and participate in the regulation of cell survival and death. Both ROS and RNS are considered to be important signals in ferroptosis.

### ROS

ROS, a byproduct of aerobic metabolism, includes superoxide anion (O_2_^•–^), hydroxyl radicals (^•^OH), hydrogen peroxide (H_2_O_2_) and singlet oxygen (^1^O_2_). The main cellular sources of ROS are mitochondrial metabolism and NADPH oxidase (NOX) at the cell membrane (Fig. 2). O_2_^•–^ is generated by the electron transport chain on the inner membrane of the mitochondria, and its production rate depends on the mitochondrial inner transmembrane potential. In the presence of mitochondrial superoxide dismutase (SOD), O_2_^•–^ is converted into H_2_O_2_, which then diffuses from mitochondria into the cytosol. At high iron concentrations favoring the Fenton reaction, H_2_O_2_ forms highly reactive O_2_^•–^ radicals. Catalase is responsible for converting H_2_O_2_ into water and oxygen. Mitochondrial ROS is important not only for apoptosis induction, but also for ferroptosis induction,^19–21^ although the molecular switches that determine the bifurcation between these two different types of cell death remain elusive. A major regulator of ATP homeostasis, AMP-activated protein kinase (AMPK), plays a dual role in ferroptosis depending on its substrate. AMPK-mediated BECN1 phosphorylation promotes ferroptosis through the inhibition of SLC7A11 activity or the induction of autophagy^71,72^ (Fig. 4a), whereas AMPK-mediated ACACA phosphorylation inhibits ferroptosis through the inhibition of fatty acid biosynthesis,^21^ indicating that energy status may affect lipid biosynthesis and peroxidation during ferroptosis.

There is a metabolic network to control ROS production during ferroptosis. In particular, carrier family 38 member 1 (SLC38A1)- and SLC1A5-mediated l-glutamine (but not d-glutamine) uptake and subsequent glutaminase 2 (GLS2, but not GLS1)-mediated glutamate production is required for cystine deprivation- or erastin-induced ferroptosis^40^ (Fig. 2). Glutamate is used to produce α-ketoglutarate (αKG) through transaminase GOT1-mediated transamination, but not glutamate dehydrogenase (GLUD1)-dependent glutamate deamination.^40^ Finally, increased αKG promotes ferroptosis through at least two mechanisms. On one hand, αKG-mediated citrate production in mitochondria is used to produce acetyl-CoA in the cytoplasm through ATP citrate lyase (ACL).^21^ Acetyl-CoA is an anabolic precursor for lipid biosynthesis by ACACA and fatty acid synthase (FASN). On the other hand, αKG stimulates dihydrolipoamide dehydrogenase (DLD) to generate mitochondrial ROS and to increase local iron level during cystine deprivation-induced ferroptosis.^73^ Thus, αKG is a metabolic intermediate for the induction of ferroptosis by producting ROS or lipid.

The VDAC family located in the outer mitochondrial membrane acts as a gatekeeper for the entry and exit of mitochondrial metabolites, thereby controlling the crosstalk between the mitochondria and the rest of the cell during oxidative stress. Initially, erastin was considered to be a direct VDAC2 and VDAC3 activator leading to an increased mitochondrial transmembrane potential^15^ (Fig. 2). Later, VDAC2 was identified as a direct target for carbonylation (a covalent modification of proteins by lipid-derived electrophils) during RSL3-induced ferroptosis.^74^ More recently, erastin was found to induce both VDAC2 and VDAC3 degradation in a NEDD4 E3 ubiquitin ligase-dependent manner in melanoma cells.^75^ These findings support the important role of VDAC in mediating mitochondrial damage (including mitochondrial ROS production) during ferroptosis.

The NOX family consists of NOX1, cytochrome B-245 beta chain (CYBB/NOX2), NOX3, NOX4, NOX5, dual oxidase 1 (DUOX1) and DUOX2. They participate in a membrane-bound enzyme complex that, together with other proteins, can transport electrons across the plasma membrane to produce superoxide and other downstream ROS. NOX1, CYBB/NOX2 and NOX4-mediated ROS production promotes lipid peroxidation in ferroptosis.^76–80^ The activity of NOXs in ferroptosis is positively regulated by a kinase (e.g., MAPK14/p38) or a binding protein (e.g., dipeptidyl peptidase 4 (DPP4/CD26))^76,80^ (Fig. 2). A full understanding of their regulatory network might pave the way for the development of effective ferroptosis inhibitors targeting these proteins.

### RNS

O_2_^•–^ can react with nitric oxide (NO) and cause nitrosative stress by forming a highly reactive RNS, peroxynitrite (ONOO^•^). The consequences of nitrosative stress may include mitochondrial dysfunction and cell death, including ferroptosis. Nitric oxide synthases (NOSs) are a family of enzymes catalyzing the production of NO from l-arginine. ONOO^•^-mediated ferroptosis is implicated in concanavalin A-induced hepatitis and this process can be inhibited by caveolin-1 (CAV1), a scaffolding protein binding to NOS3/eNOS^81^ (Fig. 2). Moreover, the inhibition of NOS2/iNOS increases the sensitivity of M1 macrophages to ferroptosis, thus increasing brain trauma damage or creating a pro-inflammatory tumor microenvironment^26^ (Fig. 2). The anti-ferroptotic activity of NOS2 may depend on the ability of NO• to inhibit the activity of ALOX15-mediated lipid peroxides.^26^ The NO precursor l-arginine can trigger pancreatitis in mice by activating ferroptosis.^82^ These findings indicate the dual role of RNS signal in ferroptosis-related sterile inflammation.

## Antioxidant system

Most classic ferroptosis activators (e.g., erastin and RSL3) are inhibitors of the antioxidant system, which is important for understanding the network of different antioxidant proteins inhibiting ferroptotic cell death.^83^

### SLC7A11

The amino acid antiporter SLC7A11/xCT/system xc^−^ is composed of two core components: the light-chain subunit SLC7A11 and the heavy-chain subunit SLC3A2 (Fig. 2). Together, they sustain the production of GSH, a master endogenous antioxidant, through serial reactions after exchanging extracellular cystine for intracellular glutamate. The synthesis of GSH depends on the availability of cysteine (generated from its precursor cystine), the level of sulfur amino acid precursors and the activity of glutamate-cysteine ligase (GCL). The inhibition of GCL by buthionine sulfoximine (BSO) induces ferroptosis on its own or enhances cellular sensitivity to ferroptosis induction by other agents.^8,33,76^ Similarly, the inhibition of SLC7A11 by small-molecule compounds (e.g., erastin) or drugs (e.g., sorafenib and sulfasalazine) or glutamate causes GSH depletion to trigger ferroptosis.^8,84^

In its reduced state, GSH acts as an electron donor and is thereby oxidized itself to the form of GSH disulfide (GSSG). The recovery of GSH from GSSG is mediated by the NADH-consuming enzyme glutathione-disulfide reductase (GSR/GR). Erastin was initially identified as a direct VDAC2/3 activator, which may induce mitochondrial dysfunction.^15^ Erastin also increases SLC7A11 expression,^8^ thus activating a feedback mechanism controlling excessive GSH consumption. The expression or activity of SLC7A11 is regulated by a variety of factors, such as TP53,^85^ NFE2L2,^86^ BRCA1-associated protein 1 (BAP1),^87^ mucin 1, cell surface-associated (MUC1),^88^ or BECN1,^71^ which in turn form a complex network to control GSH levels in ferroptosis. Overall, the inhibition of the SLC7A11 pathway is one of the most critical upstream mechanisms for inducing ferroptosis (Fig. 2).

MYB proto-oncogene, transcription factor (MYB)-mediated expression of cysteine dioxygenase type 1 (CDO1), an enzyme that converts cysteine to taurine by catalyzing the oxidation of cysteine to sulfinic acid, can promote erastin-induced death in gastric cancer cells,^89^ indicating that non-GSH-dependent cysteine metabolism is also involved in controlling ferroptosis. Cysteine can be used to synthesize CoA via the pantothenate pathway, and CoA inhibits ferroptosis caused by SLC7A11 inhibition.^90^ Thus, the metabolism of cysteine has a major impact on the propensity of cells to undergo ferroptosis.

### GPX4

GPX4 functions as a phospholipid hydroperoxidase to reduce phospholipid hydroperoxide production (AA/AdA-PE-OOH) to the corresponding phospholipid alcohol (PLOH). The expression or activity of GPX4 is controlled by selenium and GSH (Fig. 2). As GPX4 is synthesized, the nascent polypeptide chain incorporates selenium as selenocysteine (Sec), where selenium replaces the sulfur of cysteine, when a UGA stop codon is “recoded” by a Sec-tRNA and a selenocysteine insertion sequence (SECIS) within the *GPX4* mRNA. Selenium can increase the anti-ferroptosis activity of GPX4 through a selenocysteine residue at 46 (U46).^7^ At the transcriptional level, selenium-induced upregulation of *GPX4* expression by the transcription factor AP-2 gamma (TFAP2C) and specificity protein 1 (SP1) prevents ferroptosis-related cerebral hemorrhage.^91^ ACSL4 is also a direct target of SP1, which leads to intestinal I/R damage related to ferroptosis.^92^ Thus, SP1 may play a dual role in the regulation of ferroptotic tissue injury.

Mechanistically, in the catalytic cycle of GPX4, active selenol (-SeH) is oxidized by peroxide to selenic acid (-SeOH) and then reduced by GSH to intermediate selenide disulfide (-Se-SG). Finally, GPX4 is activated by the second GSH, releasing glutathione disulfide (GS-SG). During the maturation of GPX4, Sec-tRNA is one of the key regulatory elements that is positively regulated by isopentenyl pyrophosphate, a product of the mevalonate pathway. Several small-molecule compounds (e.g., RSL3, ML162, ML210, FIN56 and FINO_2_) can directly or indirectly inhibit GPX4 activity (Fig. 2), but some compounds also cause GPX4 protein degradation.^27,70,93–95^

FINO_2_ does not directly bind GPX4 or inhibit GPX4 expression.^93^ FINO_2_-induced production of mitochondrial lipid peroxidation and oxidized Fe^2+^ promotes ferroptosis in an ALOX-independent manner.^93^ Unlike other GPX4 inhibitors, ML210 is a prodrug that within cells is converted to the active form α-nitroketoxime JKE1674 that covalently inhibits GPX4.^96^ In addition to inhibiting GPX4, FIN56 also prevents CoQ10 production by binding and activating farnesyl-diphosphate farnesyltransferase 1 (FDFT1/SQS, the squalene synthase).^70^ Therefore, the knockdown of FDFT1 inhibits FIN56-induced ferroptosis in HT1080 cells.^70^ In contrast, the knockdown of FDFT1 increases ML162- and RSL3-induced ferroptosis in SQLE-deficient ALK-positive anaplastic large-cell lymphoma cells.^69^ These findings indicate that squalene metabolism may play a checkpoint role in GPX4 inhibitor-induced ferroptosis.

The mechanism of GPX4 degradation in the process of ferroptosis is still uncertain. ACACA promotes FIN56-induced GPX4 degradation in HT1080 cells.^70^ Non-oxidized dopamine is a neurotransmitter that inhibits erastin-induced GPX4 degradation in cancer cells.^97^ Activating heat shock protein family A (Hsp70) member 5 (HSPA5) expression prevents erastin-induced GPX4 degradation through the formation of HSPA5–GPX4 protein complexes.^95^ In contrast, heat shock protein 90 (HSP90)-dependent chaperone-mediated autophagy promotes erastin-induced GPX4 degradation through recognizing its KFERQ-like motif (^124^NVKFD^128^ and ^187^QVIEK^191^) in neuronal cells^98^ (Fig. 4c). Therefore, coordination between autophagy and the ubiquitin–proteasome system (UPS) is necessary to promote the degradation of GPX4.

Although GPX4 inhibition is an important downstream signal during ferroptosis, GPX4-independent ferroptosis can still occur. For example, GPX4 inhibition is not required for TP53-mediated ferroptosis, although TP53 inhibits SLC7A11 expression.^30^ The depletion of GPX4 or SLC7A11 markedly increases cellular resistance to Golgi stress-induced ferroptosis.^99^ POR is also involved in ML210-induced ferroptosis in a GPX4-independent manner.^65^ These findings demonstrate the complexity of ferroptosis using different molecular machines.

### AIFM2

Apoptosis-inducing factor mitochondria-associated 2 (AIFM2, also known as FSP1), a traditional apoptosis inducer in mitochondria, has recently been identified as an antioxidant regulator in ferroptosis, regardless of its mitochondrial function.^31,32^ N-myristoylation is required for the translocation of AIFM2 from mitochondria to the cell membrane where it catalyzes the regeneration of non-mitochondrial reduced CoQ10 using NADPH, thereby trapping lipid peroxides in a GPX4-independent manner^31,32^ (Fig. 2). In some cases, AIFM2 inhibits ferroptosis by activating ESCRT-III-dependent membrane repair instead of its oxidoreductase function.^100^ The activity of AIFM2 in ferroptosis is specifically inhibited by a small-molecule compound called iFSP1.^32^ The mevalonate pathway is the target of statins that inhibit both CoQ10 production and GPX4 function. Statins are known to inhibit selenoprotein biosynthesis, rendering them potential enhancers of ferroptosis (Fig. 2). Idebenone, a hydrophilic analog of CoQ10 with antioxidant properties, inhibits FIN56- or RSL3-induced ferroptosis, but promotes staurosporine-induced apoptosis through an as yet elusive mechanism.^70^

### CGL

Another source of cysteine is the cystathionine gamma-lyase (CTH/CGL)-mediated decomposition of cystathionine, which is a part of the transsulfuration pathway (Fig. 2). The transsulfuration pathway connects methionine and GSH biosynthesis. In the methionine cycle, methionine forms S-adenosylmethionine as a methyl donor, producing S-adenosylhomocysteine. This metabolite is converted to homocysteine, which is then recycled to yield methionine. Homocysteine has an alternative pathway that produces cystathionine by cystathionine beta-synthase (CBS) and then cysteine by CTH. Cysteine is used for GSH biosynthesis by producing γ-glutamylcysteine. This step is catalyzed by glutamate-cysteine ligase catalytic subunit (GCLC) and inhibited by BSO. The silencing of cysteinyl tRNA synthetase 1 (CARS1/CARS), an enzyme that charges tRNA^Cys^ with cysteine in the cytoplasm, inhibits erastin-induced ferroptosis associated with an increased activation of the transsulfuration pathway by the upregulation of CBS or phosphoserine aminotransferase 1 (PSAT1).^101^

### NADPH

NADPH, one of the principal reducing agents, is mainly produced by the pentose phosphate pathway (PPP) and limits the peroxidation damage caused by ferroptosis. NADPH can also be synthesized through phosphorylation of NAD by NAD kinase (NADK). The silencing of NADK reduces NADPH and enhances erastin-, RSL3- and FIN56-induced ferroptosis.^102^ HD domain containing 3 (HDDC3/MESH1) is a cytoplasmic NADPH phosphatase that, if overexpressed, causes ferroptosis.^103^ Unexpectedly, the pharmacological inhibition of PPP by 6-aminonicotinamide or knockdown of two PPP enzymes (glucose-6-phosphate dehydrogenase (G6PD) and phosphoglycerate dehydrogenase (PHGDH)) partly prevents erastin-induced ferroptosis in Calu-1 cells.^8^ In contrast, NOX-mediated NADPH oxidation promotes ferroptosis.^76–80^ Therefore, changes in the NADP/NADPH ratio may determine the sensitivity of ferroptosis, while ferroptosis-resistant cell lines may have higher basal NADPH levels or lower NADP/NADPH ratios.^102^ Many ferroptosis regulators, such as GPX4, AIFM2, NOX and POR, use the NADPH system to regulate electron transport, indicating that NADPH has an important role in chemical reactions during ferroptosis.

### AKR1C

Aldosterone reductase family 1 (AKR1), including the AKR1C and AKR1D subfamilies, is a family of aldo-keto reductase enzymes that are involved in steroid metabolism. In erastin-resistant cancer cells (DU-145, CHL-1 and SK-Mel5), an increased expression of AKR1C (including AKR1C1, AKR1C2 and AKR1C3) prevents ferroptosis by reducing the end products of lipid peroxides (AA/AdA-PE-OOHs) to the corresponding nontoxic lipid-derived alcohols (AA/AdA-PE-OHs)^84^ (Fig. 2).

### Peroxiredoxin

Peroxiredoxins (PRDXs) are a family of selenium-independent glutathione peroxidases that contribute to the suppression of ferroptosis (Fig. 2). PRDX6 is recruited to the peroxidized cell membrane after oxidative stress, where it reduces and hydrolyzes the oxidized sn-2 fatty acyl or the sn-2 ester (alkyl) bond of oxidized phospholipids. PRDX6 inhibits erastin- or RSL3-induced LOOH production and ferroptosis through its calcium-independent PLA2 activities.^104^ Similarly, PRDX5 inhibits erastin-induced ROS accumulation in HepG2 cells.^105^ Moreover, PRDX1 inhibits cumene hydroperoxide-induced ferroptosis in corneal endothelial cells.^106^

### Thioredoxin

Thioredoxin is a 12 kDa ubiquitous oxidoreductase that is central to the thioredoxin antioxidant system consisting of thioredoxin, NADPH and thioredoxin reductase. Ferroptocide rapidly induces ferroptosis-like cell death in various cancer cells through inhibiting the enzymatic activity of thioredoxin (Fig. 2), although there is no direct evidence that ferroptocide induces lipid peroxidation.^107^ The knockout of thioredoxin reductase 1 (TXNRD1) inhibits ML210-induced ferroptosis in cancer cells.^96^ Although these studies demonstrate the importance of thioredoxin in suppressing ferroptosis, they do not show that the direct activation of the thioredoxin pathway is necessary for limiting lipid peroxidation.

### GCH1

GTP cyclohydrolase-1 (GCH1) is the rate-limiting enzyme of tetrahydrobiopterin (BH_4_) biosynthesis. BH_4_ is a cofactor for several key enzymes involved in the production of neurotransmitters (e.g., dopamine) and NO. GCH1-mediated BH_4_ production causes lipid remodeling and inhibits ferroptosis by selectively preventing two polyunsaturated fatty acyl tails from consuming phospholipids^108^ (Fig. 2). Dopamine has been shown to inhibit erastin-induced ferroptosis,^97^ whereas NO-mediated ferroptosis is implicated in tissue injury.^81^ Thus, BH_4_ deficiency may play a role in the pathogenesis of ferroptosis-related disease.

## Membrane repair

One of the hallmarks of ferroptosis is membrane oxidative damage, which can be repaired by at least two mechanisms. The first is to limit lipid peroxidation by activating specific enzyme systems, such as GPX4 and AIFM2. Once the first defense system fails, cells may use vesicular transport, exocytosis and endocytosis to repair the broken membrane. The ESCRT-III machinery appears to be a common membrane repair mechanism that counteracts various forms of regulated necrosis, including necroptosis,^109^ pyroptosis^110^ and ferroptosis.^34^ ER stress-mediated Ca^2+^ influx through the Ca^2+^ channel ORAI calcium release-activated calcium modulator 1 (ORAI1) contributes to GSH depletion-induced ferroptosis in neuronal cells.^111^ At the same time, ER stress-mediated Ca^2+^ influx is a trigger for the recruitment and activation of ESCRT-III in the cell membrane during ferroptotic damage.^34^ Treatment with tauroursodeoxycholic acid (an ER stress inhibitor) or the calcium chelator BAPTA-AM limits the accumulation of the core components of ESCRT-III machinery (e.g., charged multivesicular body protein 5 (CHMP5) and CHMP6) in the plasma membrane during ferroptosis. The knockdown of CHMP5 or CHMP6 makes cancer cells sensitive to ferroptotic death, supporting the idea that the activation of ESCRT-III machinery prevents ferroptosis^34^ (Fig. 2). Understanding how cells reseal the plasma membrane after damage in ferroptosis in vivo remains a special challenge.

## Degradation systems

Macroautophagy (hereafter referred to as autophagy) and the UPS are two major intracellular degradation pathways in eukaryotic cells. They participate in the regulation of ferroptosis through the degradation of proteins and organelles, thereby controlling iron accumulation and lipid peroxidation.

### Autophagy

Autophagy is a lysosomal-dependent degradation pathway characterized by the formation of lipid-associated membrane structures that function to engulf and degrade various substrates^112^ (Fig. 4a). Oxidative stress and lipid peroxidation products (e.g., MDA and 4HNE) are powerful inducers of autophagy, and excessive autophagy promotes ferroptosis.^113,114^ Cystine deprivation results in the inhibition of mechanistic target of rapamycin kinase (MTOR), an important negative regulator of autophagy.^115^ The knockdown of key effectors of autophagy (e.g., ATG5, ATG7, ATG16L1 and BECN1) or lysosome-dependent cell death (e.g., signal transducer and activator of transcription 3 (STAT3) and cathepsin B (CTSB)) limits ferroptotic cancer cell death.^48,49,116,117^ Interestingly, the knockdown of SLC7A11 or GPX4 attenuates autophagy, thereby further protecting ferroptosis caused by Golgi stress.^99^ These findings indicate complex feedback between autophagy, ferroptosis and organelle stress.

Certain selective types of autophagy (e.g., ferritinophagy,^48,49^ lipophagy,^67^ clockophagy^118^) and chaperone-mediated autophagy^98^ promote ferroptosis in various cancer cells through the degradation of ferroptosis repressors (e.g., ferritin, aryl hydrocarbon receptor nuclear translocator-like (ARNTL/BMAL1), GPX4 and lipid droplets) (Fig. 4b, c). The core regulator of autophagosome formation, BECN1, can directly bind to SLC7A11 in an AMPK-dependent manner, causing GSH depletion to mediate ferroptosis.^71^ Rapamycin (the prototypic MTOR inhibitor) can cause pro-survival autophagy at low doses, but autophagy-dependent ferroptosis at high doses.^119^

The role of mitophagy, the selective autophagy of mitochondria, in ferroptosis remains complex. Two Parkinson’s disease-associated proteins, PTEN-induced kinase 1 (PINK1) and Parkin RBR E3 ubiquitin protein ligase (PRKN), are central to mitophagy and mitochondrial quality control. Mitophagy is implicated in HMOX1-mediated ferroptosis through increased ER stress.^120^ In PRKN-expressing HT1080 cells, the depletion of mitochondria by mitophagy fails to affect ferroptosis,^121^ which is different from another study showing that depletion of mitochondria by mitophagy inhibits ferroptosis.^19^ These controversial observations call for further mechanistic studies on mitochondria and energy metabolism in different cell types.

In general, it remains unclear how the autophagic machinery responds to ferroptosis activators to switch from a pro-survival to a lethal function. As a possibility, changes in membrane PEBP1-dependent lipid signals determine the dual function of autophagy in ferroptosis.^112,122^

### UPS

The ubiquitin–proteasome system (UPS)-mediated protein degradation also plays a broad role in ferroptosis. *BAP1* is a tumor suppressor gene that undergoes somatic mutations in multiple cancer types. BAP1-encoded deubiquitinating enzyme (DUB) reduces histone 2A ubiquitination (H2Aub) on chromatin, leading to SLC7A11 downregulation and ferroptosis.^87^ In contrast, OTU deubiquitinase, ubiquitin aldehyde-binding 1 (OTUB1) directly stabilizes SLC7A11 through protein–protein interaction, and this process is further enhanced by CD44 in lung cancer cells.^123^ Therefore, the ratio of BAP1 over OTUB1 can determine the expression of SLC7A11. NEDD4-dependent VDAC2/3 degradation and NEDD4L-mediated LTF degradation act as a feedback mechanism to limit ferroptosis in cancer cells.^41,75^

ZFP36 ring finger protein (ZFP36/TTP) is an RNA-binding protein and negative regulator of ferroptosis in hepatic stellate cells.^124^ F-box and WD repeat domain-containing 7 (FBXW7/CDC4)-mediated ZFP36 degradation inhibits autophagy-dependent ferroptosis by destabilizing *ATG16L1* mRNA,^124^ providing an example for studying the role of proteasome-dependent degradation of epigenetic regulators in autophagy-dependent ferroptosis.

The UPS is also involved in the regulation of DNA damage-induced ferroptosis. The loss of E3 ubiquitin–protein ligase ring finger protein 113A (RNF113A) triggers DNA damage-related ferroptosis.^125^ Lon peptidase 1, mitochondrial (LONP1) is an essential stress response protease that mediates mitochondrial proteostasis. LONP1-mediated degradation of transcription factor A, mitochondrial (TFAM) induces mitochondrial DNA damage, which causes ferroptosis in pancreatic cancer cells through the activation of stimulator of interferon response CGAMP interactor 1 (STING1/TMEM173)-dependent autophagy.^20^ These findings suggest that autophagy and UPS can engage in intertwined pathways to favor ferroptosis.

## Transcription factors and cofactors

Transcription factors play both transcription-dependent and -independent roles in ferroptosis.^126^ These transcription factors also regulate other types of cell death, depending on their downstream target genes and their binding partners. Here, we describe some transcription factors that are critical for ferroptosis regulation.

### NFE2L2

NFE2L2 is a master transcription factor that can coordinate the activation of a large number of cell protection genes involved in iron metabolism, oxidative defense and redox signaling during ferroptosis (Fig. 5a). After treatment with SLC7A11 inhibitors (elastin and sorafenib), the protective effect of NFE2L2 in ferroptosis was first demonstrated in HCC cells.^33^ This process is further enhanced by SQSTM1 that increases NFE2L2 protein stability through the inactivation of kelch-like ECH-associated protein 1 (KEAP1), an adapter protein of the Cullin3-based ubiquitin E3 ligase complex responsible for NFE2L2 ubiquitination.^33^

Besides quinone oxidoreductase 1 (*NQO1*) and *HMOX1*, metallothionein 1G (*MT1G*) was identified as a new NFE2L2-targeted gene, which can drive ferroptosis resistance in HCC cells.^127^ The discovery of a large number of NFE2L2 target genes (e.g., *FTH1*, *FTL*, *SLC40A1*, glutathione synthetase (*GSS*), *GCLC*, *SLC7A11*, *GPX4*, aldehyde dehydrogenase 3 family member A1 (*ALDH3A1*), *AKR1C*, sestrin 2 (*SESN2*) and ferrochelatase (*FECH*)) further demonstrate the important antiferroptotic function of NFE2L2^128^ (Fig. 5b). Of note, the NFE2L2-mediated expression of ATP-binding cassette subfamily C member 1 (*ABCC1*/*MRP1*) promotes the release of GSH into the extracellular space, which may limit the anti-ferroptotic effect of NFE2L2.^129^ Future work should focus on the identification of NFE2L2 target genes that specifically regulate ferroptosis but do not affect other types of cell death.

### TP53

*TP53*, the most commonly mutated tumor suppressor gene, plays a dual role in ferroptosis (Fig. 6). TP53 can transcriptionally inhibit *SLC7A11* expression to induce GSH depletion and ferroptosis in human osteosarcoma, or breast or lung cancer cells to suppress tumor growth.^85^ This process is enhanced by TP53 acetylation at K117, K161, and K162 (hereafter p53^3KR/3KR^), but limited by an additional TP53 acetylation at K98.^130^ TP53 also promotes ferroptosis through regulating lipid peroxidation via the transcriptional induction of *SAT1*^64^ or *GSL2*^40^ in human osteosarcoma cells or MEFs, respectively. In contrast, TP53-mediated transcriptional upregulation of cyclin-dependent kinase inhibitor 1A (*CDKN1A*/*p21*) inhibits ferroptosis in human fibrosarcoma cells in a GSH (but not cell cycle)-dependent manner.^131^

TP53 also plays a transcription-independent role to promote or inhibit ferroptosis through binding to deubiquitinase USP7 or DPP4 protein in human lung or colon cancer cells, respectively. TP53 and DPP4 are implicated in Golgi stress-induced ferroptosis.^99^ The S47 variant of TP53 (P47S) increases ferroptosis resistance in MEFs partly due to its impaired ability to induce GSL2 expression or elevated abundance of coenzyme A (CoA) and GSH production.^132^ In turn, the resulting ferroptosis deficiency causes iron to accumulate in P47S macrophages, increasing the risk of bacterial infection.^133^

MDM2 ubiquitinates TP53 for proteasomal degradation. Podocyte-specific MDM2-knockout mice develop TP53-mediated kidney injury through the activation of a mixed type of cell death, including ferroptosis.^134^ However, another study shows that MDM2 and MDM4 regulator of p53 (MDM4/MDMX) promotes ferroptosis in cancer cells through the activation of peroxisome proliferator-activated receptor alpha (PPARA/PPARα), but not TP53.^135^ A profound comprehension of the role of TP53 in ferroptosis and other types of cell death may lead to the development of novel strategies for treating and preventing cancer.

### ATFs

Ferroptosis is associated with increased ER stress, a cellular state accompanied by an accumulation of unfolded or misfolded proteins.^84^ Activated transcription factors (ATFs) (e.g., ATF3 and ATF4) are upregulated by ER stress, forming a feedback loop to regulate ferroptosis by transcriptional activation of several downstream genes. For example, *SLC7A11* is a direct negative transcriptional target of ATF3 in erastin-induced ferroptosis in human fibrosarcoma cells.^136^ ATF4 plays a dual role in inhibiting or promoting ferroptosis through the upregulation of *SLC7A11*, *SLC1A5*, DNA damage-inducible transcript 3 (*DDIT3*/*CHOP*), *HSPA5*, tribbles pseudokinase 3 (*TRIB3*) and ChaC glutathione-specific gamma-glutamylcyclotransferase 1 (*CHAC1*) in human glioblastoma, pancreatic cancer, or Burkitt’s lymphoma cells.^84,95,137,138^ The knockdown of CARS1 induces ATF4 expression through the phosphorylation of eukaryotic translation initiation factor 2 subunit alpha (EIF2S1/eIF2α), leading to the activation of the transsulfuration pathway to inhibit erastin-induced ferroptosis.^101^ However, ferrostatin-1 fails to inhibit tunicamycin (a classical ER stress inducer)-induced cell death.^99^ Although this suggests that ferroptosis may be regulated by a specific type of ER stress, it remains plausible that protein folding or the unfolded protein response can influence the redox state of mitochondria, leading to oxidative death.

### YAP1 and WWTR1

The Hippo pathway is an evolutionarily conserved signaling pathway in control of organ size, tissue homeostasis, and cancer growth. This pathway negatively regulates the activity of several transcription factors, including TEA domain transcription factor 1 (TEAD1) and transcription coactivators (e.g., yes-related protein 1 (YAP1/YAP) and WW domain containing transcription regulator 1 (WWTR1/TAZ)). The activation of the Hippo pathway promotes cadherin 1 (CDH1/E-cadherin)-dependent cell adhesion, resulting in ferroptosis resistance.^139^ In contrast, YAP1-mediated TFRC and ACSL4 expression, as well as WWTR1-mediated epithelial membrane protein 1 (EMP1) and angiopoietin-like 4 (ANGPTL4) expression, promotes ferroptosis in human renal cell carcinoma or ovarian cancer cells by increasing iron accumulation and lipid peroxidation.^77,139^ Like cell–cell contact,^140^ nectin cell adhesion molecule 4 (NECTIN4/PVRL4)-mediated cell clustering also prevents ferroptosis.^141^

### HIFs

Hypoxia-inducible factor (HIF) plays a central role in the transcriptional response to changes in oxygen supply, a key factor influencing intracellular metabolism for various pathological conditions, including tumor growth and tissue injury. HIF1 is a heterodimeric transcription factor consisting of an unstable alpha subunit (e.g., hypoxia-inducible factor 1 alpha (HIF1A)) and a stable beta subunit (e.g., aryl hydrocarbon receptor nuclear translocator (ARNT1/HIF1B)). EGLNs/PHDs exist in three isoforms, namely EGLN2/PHD1, EGLN/PHD2 and EGLN3/PHD3, responsible for the induction of Von Hippel-Lindau tumor suppressor (VHL)-dependent HIF1A degradation. EGLNs are a key target for iron chelators (e.g., deferoxamine), which increase HIF1A levels to protect against ischemia reperfusion injury.^142^ Stabilization of HIF1A by hypoxia or cobalt chloride promotes fatty acid uptake and lipid storage by transcriptional upregulation of fatty acid-binding protein 3 (FABP3) and fatty acid-binding protein 7 (FABP7), and ultimately inhibits ferroptosis in human fibrosarcoma or lung cancer cells through increasing lipid droplet formation.^118^ Carbonic anhydrase 9 (*CA9*), a classical HIF1A target gene, promotes malignant mesothelioma resistance to ferroptosis and apoptosis under hypoxia.^143^

Unlike HIF1A, endothelial PAS domain protein 1 (EPAS1, also known as HIF2A) appears to promote ferroptosis in clear cell carcinoma cells through transcriptional upregulation of hypoxia-inducible lipid droplet-associated (HILPDA/HIG2),^144^ a regulator of enriched lipids that contains polyunsaturated fatty acyl side chains.^144^ VHL-mediated β-oxidation or mitochondrial ATP synthesis inhibits ferroptosis in clear cell renal cell carcinoma through a GSH-independent manner.^145^ These findings indicate that HIF-mediated ferroptosis regulation is tumor type-dependent.

### HSF1

Heat shock transcription factor 1 (HSF1)-mediated heat shock response increases heat shock protein expression to protect against injury caused by environmental stresses (e.g., increased temperature, oxidative stress and heavy metals). Heat stress or HSF1-mediated expression of HSPB1 increases the resistance of cancer cells to ferroptosis through the inhibition of iron uptake.^42^ The cold stress-mediated activation of the mitogen-activated protein kinase kinase kinase 5 (MAP3K5/ASK1)-MAPK14/p38 pathway also suppresses erastin- and RSL3-induced cell death.^146^ It is unclear whether different profiles of fat consumption under cold and heat stress may affect the propensity of cells to undergo ferroptosis.

### ARNTL

The circadian rhythm is an endogenous oscillation mechanism that activates two important transcription factors, ARNTL and clock circadian regulator (CLOCK). *EGLN2* is a negative target gene of ARNTL in ferroptosis.^118^ ARNTL-mediated EGLN2 downregulation blocks ferroptosis in cancer cells through the activation of the HIF1A pathway.^118^ The conditional knockout of ARNTL disrupts the circadian rhythm of the pancreas and increases the susceptibility to pancreatitis associated with ferroptosis by inhibiting the expression of various antioxidants or membrane repair genes (e.g., *SLC7A11*, *GPX4*, superoxide dismutase 1 (*SOD1*), thioredoxin (*TXN*), *NFE2L2* and *CHMP5*).^82^ Melatonin helps maintain the body’s normal circadian rhythm and inhibits heme-induced platelet activation and ferroptosis.^147^ Altogether, the circadian rhythm may systemically control the ferroptotic response throughout the body.

### JUN

The transcription factor JUN is a basic leucine zipper transcription factor that can regulate gene transcription in various biological processes. The overexpression of JUN-WT, but not its S73A mutant (a non-O-GlcNAcylated form of JUN), induces the transcription of *PSAT1* and *CBS* to suppress ferroptosis, suggesting that O-GlcNAcylated JUN-mediated GSH synthesis is necessary for ferroptosis resistance.^148^ However, the regulation of O-GlcNAcylated JUN remains a matter of uncertainty.

### BACH1

The BTB domain and CNC homolog 1 (BACH1) mainly act as a transcriptional repressor by heterodimerizing with MAF BZIP transcription factor (MAF) protein and binding MAF recognition elements in the promoters of targeted genes. BACH1 promotes ferroptosis by repressing the transcription of a subset of protective genes, such as *GCLM*, *SLC7A11*, *FTH1*, *FTL* and *SLC40A1*.^149^ Because these genes are typical NFE2L2 target genes, functional interactions between NFE2LA and BACH1 may occur in ferroptosis.

## Epigenetic regulation

There is mounting evidence that epigenetic regulation impacts the propensity of cells to undergo ferroptosis through pathways that are closely related to the activity of transcription factors as well as posttranscriptional mechanisms.

### Chromatin remodeling

Chromatin remodeling is a dynamic modification of chromatin structure that allows the access to concentrated genomic DNA to regulate transcription machinery. Helicase, lymphoid-specific (HELLS/LSH), a chromatin remodeling factor, is a ferroptosis repressor through chromatin modification of SCD and fatty acid desaturases 2 (FADS2).^150^ SCD, an enzyme that catalyzes the rate-limiting step in the synthesis of MUFA, has the ability to inhibit ferroptosis in ovarian cancer cells.^62^ The expression of HELLS is positively regulated by transcription factor MYC and negatively regulated by HIF1A.^150^ This allows HELLS to respond quickly to changes in hypoxic environments and dynamically regulate many target genes.

### miRNAs

MicroRNAs (miRNAs) are small non-coding RNAs of approximately 22 nucleotides that regulate gene expression at the posttranscriptional level. MIR137, MIR9 and MIR103A1/MiR-103a-3p inhibit erastin-induced ferroptosis in cancer cells by directly inhibiting the expression of the metabolism-related genes *SLC1A5*, *GOT1* and *GLS2*, respectively. MIR17HG/MiR-17-92 inhibits ferroptosis in endothelial cells by inhibiting the expression of TNF alpha-induced protein 3 (TNFAIP3/A20), a positive regulator of ACSL4.^151^ MIR7-1/MiR-7-5p also causes ferroptosis resistance through the downregulation of SLC25A37/mitoferrin (a mitochondrial iron importer for the synthesis of mitochondrial heme and iron–sulfur clusters). Moreover, ZFP36 promotes *ATG16L1* mRNA decay, leading to resistance to autophagy-dependent ferroptosis.^124^

In addition to inhibiting ferroptosis, some miRNAs promote ferroptosis. For example, MIR4715/MiR-4715-3p promotes ferroptosis by inhibiting aurora kinase A (AURKA) expression. MIR30B/MiR-30b-5p inhibits the expression of paired box 3 (PAX3) and SLC7A11, thereby causing ferroptosis in trophoblastic cells. However, our understanding of when and how miRNAs exert their functions in the induction of gene expression in ferroptosis is still limited.

### LncRNAs

Long non-coding RNAs (lncRNAs) have more than 200 nucleotides. The anticancer reagent XAV939 inhibits the expression of SLC7A11, which is related to lncRNAs enriched in a ferroptosis pathway.^152^ The cytosolic lncRNA P53RRA competitively inhibits the binding of Ras-GTPase-activating protein-binding protein 1 (G3BP1) to TP53, which causes TP53-mediated cell cycle arrest, apoptosis and ferroptosis. The interaction between the nuclear lncRNA LINC00336 and ELAV-like RNA-binding protein 1 (ELAVL1) inhibits ferroptosis through promoting CBS expression in lung cancer cells.^153^ However, ELAVL1-mediated *BECN1* mRNA stability promotes autophagy-dependent ferroptosis and limits liver fibrosis,^72^ indicating a dual role for ELAVL1 in ferroptosis. LncRNA GABPB1-AS1 inhibits the translation of GA-binding protein transcription factor subunit beta 1 (GABPB1), leading to PRDX5 downregulation and subsequent ferroptosis in HepG2 cells.^105^ Thus, the lncRNAs exhibit their biological functions by acting as cis- or trans-regulators in ferroptosis.

### CircRNAs

Circular RNAs (circRNAs) are closed lncRNAs in which the 5’ and 3’ ends are covalently linked by reverse splicing of exons from a single pre-mRNA. The circRNA tau tubulin kinase 2 (circ-TTBK2) inhibits erastin-induced ferroptosis in glioma cancer cells through increasing integrin subunit beta 8 (ITGB8) expression, while MiR-761 inhibits ITGB8 expression.^154^ circ-0008035 positively regulates the expression of EIF4A1 through sponging MIR599, inhibiting ferroptosis in gastric cancer cells.^155^ The biogenesis of circRNA must continue to be studied to uncover the rules that control the production of circRNA during ferroptosis.

### Histone modifications

The deubiquitination of H2Aub suppresses, whereas monoubiquitination of histone H2B on lysine 120 (H2Bub1) promotes, the expression of SLC7A11 during ferroptosis through an epigenetic mechanism coupled to the UPS pathway.^87^ HIC ZBTB transcriptional repressor 1 (HIC1) and hepatocyte nuclear factor 4 alpha (HNF4A) are identified as pro- and anti-ferroptosis transcription factors, respectively, in HCC cells by binding to histone acetyltransferase KAT2B to affect GSH production.^156^ The pharmacological inhibition of epigenetic regulator bromodomain-containing 4 (BRD4) by JQ1 induces ferritinophagy and ferroptosis, which is linked to the downregulation of GPX4, SLC7A11 and SCL3A2 and the upregulation of ferritinophagy-related genes.

### DNA methylation

The MUC1 transmembrane glycoprotein is overexpressed in many cancers. MUC1 can bind to the CD44 variant to enhance the stability of SLC7A11, thereby inhibiting erastin-induced ferroptosis in triple-negative breast cancer cells.^88^ In turn, silencing SLC7A11 increases H3K9 methylation (e.g., H3K9me2 and H3K9me3) of the MUC1 promoter, which may further affect GSH production during ferroptosis.^88^ The histone demethylase KDM3B prevents ferroptosis in HT1080 cells by upregulating SLC7A11 in an ATF4-dependent fashion. How this is achieved without affecting the expression of other ferroptosis-relevant genes is an open question.

## Assays for ferroptosis

There are a number of methods to measure ferroptotic response related to iron and lipid metabolism. Although it is relatively easy to measure ferroptosis in vitro, in cultured cells, it is challenging to monitor ferroptotic response in vivo.

### Iron

Iron in biological samples can be easily assayed by commercially available kits. In diagnostic laboratories, Prussian blue staining is a common method used by pathologists to detect iron in biopsy specimens. The fluorescent probes Ferrum 430, Ferrum 560, and Ursa 520-R can be used to quantify the amount of Fe^3+^, but not the amount of Fe^2+^ and other metal ions. In addition, the green fluorescent heavy metal indicator Phen Green SK can be used to detect the level of Fe^2+^, but this probe can also react with a variety of metal ions, such as Cd^2+^, Co^2+^, Ni^2+^ and Zn^2+^. FIP-1, an endoperoxide reactivity-based fluorescence resonance energy transfer probe, can be used to test labile iron pools in living cells.^157^ Mito-FerroGreen can be used for the detection of Fe^2+^ in mitochondria. Staining TFRC with antibodies (e.g., 3F3-FMA) can test for ferroptosis activity in cell culture and tissues.^158^ The expression of TFRC may be tissue-specific, especially in iron-rich tissues.

### Lipid ROS

Several image or biochemistry methods can be used to detect the presence of lipid ROS. These include assays for the direct quantification of LOOHs as well as assays that quantify end-product reactive aldehydes (e.g., MDA or 4HNE) or antioxidants (e.g., GSH, CoQ10 and NADPH). Among them, the fluorometric probes C11-BODIPY, Click-It LAA and LiperFluo (a perylene derivative containing oligooxyethylene) are the most widely used methods for indexing lipid peroxidation during ferroptosis. C11-BODIPY and LiperFluo can react with peroxyl radicals, whereas LiperFluo (but not C11-BODIPY) interacts with (phospho)LOOHs.^24^ MitoPerOx, MitoPeDPP and MitoCLox are fluorescent probes for testing mitochondria-targeted lipid peroxidation, while MitoSOX is among the most commonly used probe for detecting mitochondrial superoxide in cells.

Lipid oxidation products contained in cells or tissue samples can be identified and quantified by mass spectrometry. In addition, monoclonal antibodies specific for MDA or 4HNE can be used to detect lipid peroxidation in tissue samples by immunohistochemistry. Decreased capacities of cystine uptake and CoQ10 production, and increased capacities of NADPH oxidation and GSH depletion are associated with increased ferroptosis in various conditions.^27,84^ Increased ACSL4 and PTGS2 expression are related to increased lipid peroxidation-dependent ferroptosis in vivo. CellROX Green Reagent, which preferentially binds to oxidized DNA, can be used to measure DNA damage.^159^ A quinoxalinone-based fluorescent probe (QS-4) can be used to monitor ferroptosis in living cells.^160^ Moreover, the reversible reaction-based fluorescent probe RealThiol can quantitatively monitor real-time GSH dynamics in living cells during ferroptosis.^161^ The specificity and sensitivity of these probes need further study.

## Implications of ferroptosis in disease

Excessive or defective ferroptosis can contribute to pathological cell loss, as well as to malignant processes. Here, we summarize the effects of ferroptosis on various pathologies and disease states.

### Cancer

The complex role of ferroptosis in tumor treatment and tumorigenesis is not only affected by oncogenes and tumor suppressors, but also by the tumor microenvironment. Targeting ferroptotic pathways are implicated in chemotherapy, immunotherapy and radiation therapy.

#### Gene mutation

It has been speculated that the induction of ferroptosis may be used as a targeted therapy for cancers harboring oncogenetic RAS mutations.^8^ However, both RAS-dependent and -independent pathways facilitate ferroptotic cancer cell death.^27,162,163^ RAS mutations can even cause ferroptosis resistance in some cases, such as in human rhabdomyosarcoma cells.^164^ DNA damage accumulates in ferroptosis-sensitive p53^3KR/3KR^ cells, which leads to aneuploidy, a hallmark of cancer-related genomic instability.^165^ The sensitivity of ferroptosis is also affected by mutated epidermal growth factor receptor (EGFR)^78^ or mutated IDH1.^166^ Overall, the function of gene mutations in ferroptosis is context-sensitive.

#### Epithelial–mesenchymal transition

The epithelial–mesenchymal transition (EMT), a process in which epithelial cells acquire mesenchymal features, seems to be important for ferroptosis induction. Both GPX4 inhibitors (e.g., RSL3, ML210 and ML162) and statins (e.g., fluvastatin, lovastatin acid and simvastatin) reportedly may target the mesenchymal state for cancer therapy.^167^ In contrast, increased cell adhesion confers resistance to ferroptosis, which is mediated by CDH1/E-cadherin,^167^ the Hippo pathway,^77,139^ or integrins (e.g., ITGA6 and ITGB4).^168^ Therefore, inducing ferroptosis may only be effective for certain types of cancer, requiring careful evaluation of cell type- and differentiation state-related signaling pathways that may dictate sensitivity and resistance to ferroptosis.

#### Tumor treatment

**Fig. 7:**
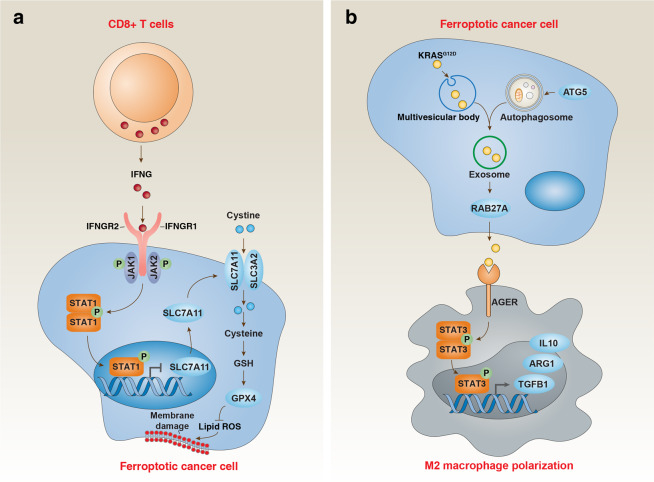
Dual role of ferroptosis in tumor immunity. **a** CD8^+^ T cell-mediated IFNG release inhibits SLC7A11 expression in cancer cells through activation of the STAT1 pathway, thereby inducing tumor cell ferroptosis. **b** Ferroptotic cancer cell-mediated KRAS^G12D^ release increases M2 macrophage polarization through activation of the STAT3 pathway, thereby limiting antitumor immunity.

Erastin, GPX4 inhibitors and some drugs (e.g., sorafenib, sulfasalazine, artesunate, cisplatin, ibuprofen, lanpersone and DHAN) trigger ferroptosis in various types of cancers, but other types of cell death contribute to their anticancer activity as well. For example, DHAN and artesunate induce both ferroptosis and apoptosis. Inducing ferroptosis also shows a benefit in enhancing the anticancer activity of checkpoint blockade (e.g., anti-CD274/PD-L1 and anti-cytotoxic T lymphocyte-associated protein 4 (CTLA4)) and radiotherapy.^169,170^ Mechanistically, CD8^+^ T cell-mediated IFNG release inhibits SLC7A11 expression in cancer cells, thereby inducing tumor cell ferroptosis^169^ (Fig. 7a). While signal transducer and activator of transcription 1 (STAT1) is required for interferon gamma (IFNG/IFNγ)-induced SLC7A11 suppression,^169^ ATM is required for radiotherapy-mediated SLC7A11 downregulation.^170^ Ionizing radiation increases the expression of ACSL4 and GPX4, thus activating a feedback loop to control ferroptotic death.^171^ Ferroptosis activators can also cause DNA damage and cell death in normal bone marrow cells as well as in various tissues, indicating potentially deleterious side effects.^172^ For this reason, strategies for therapeutic ferroptosis induction must be carefully evaluated to identify optimal compounds, doses and schedules, as well as correct neoplastic indications.

#### Tumorigenesis and development

Ferroptosis has been reported to either inhibit or promote tumorigenesis in different models. The conditional knockout of SLC7A11 in the pancreas delays KRAS^G12D^TP53^R172H^-induced pancreatic cancer growth, partly through cysteine depletion-induced ferroptosis.^90^ However, some studies indicate that cysteine starvation alone is not sufficient to induce ferroptosis because cysteine and GSH have compensatory roles in the prevention of ferroptosis.^115^ Whether an abundance of GSH directly impacts tumorigenesis requires further investigation. The lymphatic system can enhance melanoma metastasis by providing ACSL3-dependent MUFA production to limit ferroptotic cancer cell death.^173^

It should be noted that ferroptosis may have paradoxical pro-tumorigenic effects. Ferroptosis-mediated DAMP release may promote tumor progression through sustaining an inflammatory tumor microenvironment. In this context, KRAS^G12D^ protein is a DAMP released from ferroptotic cancer cells and is taken up by macrophages through AGER, resulting in STAT3-dependent macrophage polarization and subsequent pancreatic tumor growth^174^ (Fig. 7b). The interaction between ferroptosis, inflammation and immunity could stage-dependently affect the outcome of cancer development and needs to be considered when designing new therapeutic approaches.

### Nervous system

Brain iron accumulation is closely related to neurological diseases and hemorrhagic stroke. The lysis of red blood cells after intracerebral hemorrhage leads to the release of hemoglobin, which is further broken down into heme and its oxidized form, hemin. Hemoglobin- and hemin-induced toxicity reportedly involve both ferroptosis and necroptosis.^175,176^ Iron overload-induced ferroptosis is implicated in Pelizaeus-Merzbacher disease caused by mutations in proteolipid protein 1 (PLP1)^177^ or neuroferritinopathy.^178^ The ferroptosis inhibitor SRS11-92 is highly effective in protecting human primary fibroblasts from cell death induced by frataxin depletion, indicating that targeting ferroptosis might be useful for the treatment of Friedreich ataxia, pending confirmation in animal models.^179^

Neurodegenerative diseases, such as Huntington’s disease (HD), Alzheimer’s disease (AD) and Parkinson’s disease (PD), are incurable conditions that cause the progressive degeneration and death of neurons. Ferrostatins (e.g., ferrostatin-1 and SRS11-92) restore the number of healthy neurons in an HD model^180^ and protect oligodendrocytes against cysteine deprivation-induced injury.^180^ Iron chelators (e.g., DFO, M30 and alpha-lipoic acid) and ferrostatin-1 also prevent the development of AD and PD by increasing HIF1A stability in the brain, which inhibits neuronal death, including ferroptosis.^181–183^ The exact impact of specific iron metabolism genes on neurodegenerative diseases under oxidative stress appears to be stimulus-dependent.

### Digestive system

Mice with a conditional hepatic knockout of GPX4 die before birth,^184^ indicating a potential role of lipid peroxidation in liver development. Liver damage caused by drugs (e.g., acetaminophen and phenylhydrazine), ischemia/reperfusion (I/R), ethanol, a high-iron diet, or genetic manipulation (e.g., overexpression of adipose tissue-specific lipin 1 (LPIN1)) is partly inhibited by ferrostatin-1. Ferroptosis is also implicated in nonalcoholic steatohepatitis induced by a choline-deficient and ethionine-supplemented diet or methionine-choline-deficient diet.^185^ Conversely, induced ferroptosis may be beneficial in the treatment of certain liver diseases, such as liver fibrosis or parasite infections of Plasmodium at the liver stage.^72,186^

The heterozygous knockout of GPX4 in intestinal epithelial cells increases the inflammatory bowel disease (IBD) in mice caused by a PUFA-rich Western diet (containing 10% fish oil with omega-3 and omega-6 PUFAs).^59^ The inhibition of ferroptosis by liproxstatin-1 and rosiglitazone (an ACSL4 inhibitor) also reduces intestinal I/R injury.^92^ It remains to be seen whether chronic inflammation mediated by ferroptosis is related to colon cancer.

Ferroptosis is also involved in pancreatic disease. The conditional knockout of the circadian rhythm gene *ARNTL* increases l-arginine-induced acute pancreatitis and HMGB1 release, which can be suppressed by liproxstatin-1.^82^ Thus, perturbation of the circadian clock exacerbates the risk of sterile inflammation due to increased oxidative stress and ferroptotic damage.

### Respiratory system

Transforming growth factor β1 (TGFB1/TGFβ1) is the main pro-fibrotic cytokine in the progression of idiopathic pulmonary fibrosis, which induces lung fibroblasts to differentiate into myofibroblasts that produce high levels of collagen, resulting in a loss of lung elasticity and function. TGFB1 suppresses GCL expression and increases lipid peroxidation in mouse lungs,^187^ indicating that ferroptosis may be involved in pulmonary fibrosis. Liproxstatin-1-mediated NFE2L2 activation prevents radiation-induced pulmonary fibrosis,^188^ suggesting a protective role for NFE2L2 in limiting lung fibrosis.

Autophagy-dependent ferroptosis is implicated in chronic obstructive pulmonary disease (COPD) caused by air pollution. For example, in *GPX4*^*+*/*−*^ mice, exposure to cigarette smoke increases the infiltration of immune cells (e.g., macrophages and lymphocytes), DAMPs (e.g., HMGB1) and cytokine (e.g., IL1A, IL33 and TNF) production in bronchoalveolar lavage fluid.^189^

ALOX-dependent ferroptosis also plays a role in lung infection. The formation of PEBP1–ALOX15 complexes may mediate oxidative injury and asthma-related airway inflammation.^25^ Interestingly, *P. aeruginosa* can release ALOX15 to induce ferroptosis in bronchial epithelial cells, indicating that pathogen-mediated ALOX15 production mediates host cell death and infection.^190^

### Urinary system

The kidneys are the main organs responsible for drug excretion. Increased expression of ACSL4 is associated with the severity of acute kidney injury (AKI).^94^ Ferrostatins (e.g., ferrostatin-1, SRS11-92 and SRS16-86) protect AKI induced by rhabdomyolysis, hydroxyquinoline, ferrous ammonium sulfate, nephrotoxic folic acid, I/R and oxalate, thus reducing cytokine production (e.g., IL33, TNF and MCP1) in mice. Similarly, GPX4 depletion in the kidney causes AKI in a ferroptosis-dependent manner.^16^ In contrast, growth factor, augmenter of liver regeneration (GFER/ALR) has a protective effect on I/R-induced kidney injury through binding to GPX4, further underscoring that GPX4 is a negative regulator of AKI.

Of note, mixed cell death is common in AKI. The depletion of macrophage migration inhibitory factor (MIF), a regulator of inflammatory and immune responses, increases I/R-induced AKI accompanied by necroptosis and ferroptosis.^191^ Ferrostatins can further enhance the protective effect of necroptosis inhibitors on renal I/R injury. TP53-mediated mixed cell death, including apoptosis, pyroptosis, pyronecrosis, necroptosis, ferroptosis, and parthanatos, is involved in MDM2 depletion-induced kidney injury in *Mdm2*^*flox*/*flox*^;*Nphs2-cre*^+^ mice.^134^ The extent of the contribution of ferroptosis to AKI caused by different risk factors is still unknown.

### Reproductive system

Testicular I/R injury induces cell death in germ cells and support cells, while inhibiting cell death can improve testicular function. Treatment with oxygen-glucose deprivation/reoxygenation or arsenite induces ferroptosis in testicular cells, which is involved in the activation of the ACSL4-ALOX15 pathway.^192^ In contrast, treatment with NAC, liproxstatin1 and iron chelators inhibits ferroptosis in testicular cells. In women with preeclampsia, increased iron and lipid ROS in the placenta coincide with ferroptosis in trophoblasts characterized by the downregulation of SLC7A11 and PAX3.^193^ These findings suggest that inhibiting ferroptosis may be a potential strategy for treating reproductive toxicity caused by drugs or stress.

### Circulatory system

Heart injury (e.g., caused by myocardial infarction, I/R, or anthracycline-based chemotherapy) is associated with an increased ferroptotic response with reduced GPX4 and increased iron accumulation.^194^ The activation of BACH or toll-like receptor 4 (TLR4) signaling causes iron overload, inflammation, or autophagy, which leads to ferroptosis-related heart damage in mice.^79,149,195^ Dexrazoxane (an iron chelator) and ferrostatin-1 prevent doxorubicin- and I/R-induced heart damage by inhibiting mitochondrial iron-dependent lipid peroxidation and ferroptosis.^196^ At the genetic level, the activation of the MTOR or ectonucleotide pyrophosphatase/phosphodiesterase 2 (ENPP2) pathway inhibits ferroptosis-mediated cardiomyocyte injury. Thus, ferroptosis mediates acute myocardial injury.

### Immune system

T cell-specific Gpx4-deficient mice (T^ΔGpx4/ΔGpx4^) were first used to study ferroptosis in immunity.^35^ Due to T-cell death by ferroptosis, T^ΔGpx4/ΔGpx4^ mice are more sensitive to viral and parasitic infections.^35^ GPX4 is also important for the development of B1 and marginal zone B cells. B cell-specific Gpx4-deficient mice show an impaired IgM antibody response to *S. pneumoniae* infection.^197^ However, by inhibiting the expression of the bone morphogenetic protein family, erastin induces the proliferation and differentiation of human peripheral blood mononuclear cells into B cells and natural killer cells.^198^ These findings indicate a role of ferroptosis in regulating immune cell development, differentiation and viability.

Myeloid cells, including macrophages and monocytes, play a key role in innate immunity. Hemoglobin, heme and hemin produced by old red blood cells trigger ferroptosis in macrophages, monocytes and platelets, thereby regulating immune response.^199^ Myeloid cell-specific Gpx4-deficient mice are sensitive to lipopolysaccharide (LPS)- or cecal ligation and puncture-induced septic death in a ferroptosis-independent manner.^200^ Uncontrolled macrophage death by ferroptosis can lead to tissue damage, thus augmenting *M. tuberculosis* dissemination.^201^ Ferroptosis also regulates macrophage polarization through cholesterol metabolism or fatty acid oxidation, resulting in a unique functional phenotype in infection and immunity.^26,174^

## Conclusions and perspectives

Over the past 5 years, we have witnessed an outbreak of ferroptosis research in biomedicine, which has resulted in the publication of over 1000 papers. In general, ferroptosis is considered to be a form of regulated necrosis, which is strictly controlled at multiple levels.^202,203^ Ferroptosis can occur by targeting two main pathways, the extrinsic or transporter (e.g., SLC7A11, SLC38A1, NOXs and TFRC)-dependent pathway and the intrinsic or enzyme (e.g., ACSL4, ALOXs, GPX4, POR, GCH1, NOS and AIFM2)-regulated pathway. At the core of the process, PUFAs and PUFA-containing lipids are highly sensitive to oxidation by enzymes (e.g., ALOX and POR) and non-enzymatic reactions (e.g., using an iron-dependent Fenton reaction). Many pharmacological agents or genetic manipulations have been used to modulate the ferroptotic response in the context of an array of different disease models, often attenuating morbidity and mortality. Colliding with this enthusiasm is the fact that there are many unresolved problems in ferroptosis research that must be tackled in the future.

### What are the thresholds of damage required to induce ferroptosis?

Under physiological conditions, iron and ROS are important for cell proliferation and signal transduction. Under pathological conditions, the initial reaction to stressful stimuli usually subserves the purpose to defend cells against insults, often resulting in a hormetic response. Once a threshold is trespassed, iron overload and oxidative damage can cause ferroptotic cell death. Multiple oxidation and antioxidant systems can be activated simultaneously and run in parallel to adjust this threshold, which is associated with metabolic reprogramming of the affected cell. The level of iron and ROS needed for trespassing the point of no return during ferroptosis remains unknown, as does whether the kinetics of iron and redox stress determine the outcome. We also must determine whether there are specific metabolic checkpoints for modulating oxidation and antioxidant reactions during ferroptosis.

### What is the final effector of ferroptotic cell death?

Iron accumulation and lipid peroxidation may be considered as intermediate events but they are not the final executors of ferroptosis. Not all damage caused by lipid peroxidation results into ferroptotic cell death. Key regulators of ferroptosis also have activity in regulating other types of cell death. For example, GPX4 also inhibits apoptosis,^204^ necroptosis^205^ and pyroptosis^200^ in response to various tissue injuries. The activation of SLC7A11 also avoids apoptosis under oxidative stress.^206^ Increased lipid peroxidation may be a common event in distinct cell death pathways, which then however, would rely on different last-stage effectors. Hypothetically, like other types of regulated necrosis, ferroptosis may require the activation of yet-to-be-identified pore-forming proteins to cause membrane rupture. Alternatively, lipid peroxidation-mediated reduction in membrane fluidity or structural changes (e.g., reduced membrane thickness) may disrupt the function of the plasma membrane as a selective barrier. Finally, excessive lipid oxidation products (including proteins and DNA) may directly cause cytotoxicity.

### How do we define the interaction and conversion between ferroptosis and other types of cell death?

Different types of cell death usually occur downstream of a common stress response (or its failure) and share similar initial signals and molecular regulators including at the level of ER stress, redox stress, mitochondrial dysfunction and autophagy. Although the depletion of BAX/BAK1 does not affect ferroptosis,^8^ other BCL2 families, such as BID, BCL2 and BCL2-binding component 3 (BBC3/PUMA), may be involved in regulating the transformation between ferroptosis and other types of cell death (e.g., apoptosis and anoikis).^207,208^ The inhibition of ferroptosis may cause cells to engage in different lethal subroutines (instead of avoiding cell death as such). Conversely, the interruption of apoptotic or necroptotic pathways may shift cells towards a ferroptotic cell death pathway. However, the mechanisms that allow cells to “choose” among different cell death modalities are still enigmatic. The definition of cell death may require comprehensive consideration of the initial signal, intermediate mediators, as well as end effector molecules, which we might call the “cell death code”. Different types of cell death may share parts of the code, but the entire code should be distinct for each lethal subroutine. In this scenario, the specificity of the elements of the current ferroptosis code remains elusive.

### How do we evaluate the pathological role of ferroptosis in a disease?

In many cases, researchers have concluded that excessive cell death by ferroptosis is involved in pathogenic processes because ferrostatin-1 or liproxstatin-1 prevents tissue damage. Although these inhibitors are safe in preclinical animal studies, they are essentially antioxidants and may inhibit other ROS-dependent forms of cell death. More selective and safer ferroptosis drugs specifically targeting the ferroptosis machinery are needed to evaluate the pathophysiological effects of ferroptosis in the future. The conditional knockout of GPX4 or SLC7A11 in mouse organs does not prove that ferroptosis is pathogenic, because the deletion of GPX4 and SLC7A11 causes a surge in oxidative stress that may promote non-ferroptotic death. Unlike mice with a deadly global GPX4-deficiency, SLC7A11-deficient mice develop normally, suggesting that the two genes have distinct functions in regulating oxidative stress.^209^ Under normal circumstances, the depletion of SLC7A11 does not induce ferroptosis in mice.^210^ Keratinocytes in mice with GCL deficiency display GSH depletion, cell death and inflammation, mainly in the form of apoptosis.^211^ Therefore, distinguishing different types of oxidative cell death in the body remains a challenge. We also need to assess the short-term and long-term effects of ferroptosis on immune cell function, which may depend on the identification of ferroptosis-specific DAMPs. Hence, there is an urgent need to identify biomarkers that inform us on the proclivity of cells to undergo ferroptosis and the lethal ferroptotic event itself, as well as on its downstream consequences.

In summary, we are at the dawn of a new era of ferroptosis research. Since one key cannot unlock every lock, it will be important to decipher context-specific regulatory mechanisms that may ultimately facilitate tissue-specific or cell type-specific interventions on the ferroptotic process in multiple distinct diseases.^212^ These challenges will require ever more specific drugs, sophisticated preclinical models, and innovative technology.

## Supplementary information

Supplemental Information
