# Supplementary material for: Ferroptosis: molecular mechanisms and health implications
Source: Cell Res. 2020 Dec 2;31(2):107–25. doi: 10.1038/s41422-020-00441-1 (PMC8026611; doi:10.1038/s41422-020-00441-1)
Supplement: Supplementary file 1 — Supplemental Information [file 41422_2020_441_MOESM1_ESM.pdf]

Table S1. Main pharmacological modulators of ferroptosis

**Inducers/ sensitizers**	**Class**	**Example**	**Refs**
	AKR1C1 inhibitor	Medroxyprogesterone	[1]
	ALDH inhibitor	Oxyfedrine, dyclonine	[2]
	Autophagy inducer	Loperamide, pimozide, STF-62247, BAY 87-2243, amentoflavone, zalcitabine	[3-5]
	CoQ10 synthesis inhibitor	FIN56, iFSP1	[6, 7]
	Golgi stress inducer	AMF-26/M-COPA, golgicide A, brefeldin A	[8]
	GPX4 inhibitor	RSL3, ML162, ML210, DPIs, FIN56, $FINO_2$	[9-11]
	GSH synthesis inhibitor	Buthioninesulfoximine (BSO), CH004, cyst(e)inase	[11]
	HIF1A and CA9 inhibitor	Chetomin, KC7F2, S4, U104	[12, 13]
	HMGCR inhibitor	Statins (e.g., cerivastatin and simvastatin)	[6]
	Iron-containing product or inducer	Hemin, hemoglobin, ironomycin, Fe-8HQ, withaferin A, $(NH_4)_2Fe(SO_4)_2$	[14-16]
	NFE2L2 inhibitor	Trigonelline, brusatol, propargylglycine, ibuprofen	[17, 18]
	SCD inhibitor	MF-438, CAY10566, A939572	[19]
	System xc^- inhibitor	Erastin, sulfasalazine, sorafenib, imidazole ketone erastin (IKE), erastin2, glutamate	[20, 21]
	TXN inhibitor	Ferroptocide	[22]
	VDAC2/3 activator	Erastin, RSL5	[9, 20]
**Inhibitors**	ACSL4 inhibitor	Rosiglitazone, triacsin C	[23, 24]
	ALOX inhibitor	Zileuton, MK886, PD146176, BWA4C, baicalein, cinnamyl-3,4-dihydroxya-cyanocinnamate (CDC), AA-861, LOXBlock-1	[25-27]
	Broad-spectrum antioxidant	Butylated hydroxyltoluene (BHT), vitamin E, α-tocopherol, β-carotene, n-acetylcysteine (NAC), β-mercaptoethanol, phenoxazine, GSH, trolox, CoQ10, idebenone, curcumin, selenium, melatonin, edaravone, quercetin	[21, 28-30]
	Calcium blocker	2-aminoethoxydiphenyl borate (2-APB), cobalt chloride, apomorphine, LY83583	[31, 32]
	DPP4 inhibitor	Vildagliptin, alogliptin, linagliptin	[33]

	Glutamine metabolism inhibitor	L-g-glutamyl-p-nitroanilide (GPNA), aminooxyacetic acid (AOA), compound 968	[21, 34]
	Iron chelator or inhibitor	Deferoxamine (DFO), 2,2-bipyridyl, ciclopirox olamine (CPX), deferiprone, pioglitazone	[9, 21, 35]
	Kinase inhibitor	U0126 (MEK), SU6656 (SRC), Flt3 inhibitor III (FLT3), MJ33 (PLA2), Gö6983 (PKC), SP600125 (MAPK8/JNK), SB202190 (MAPK14/p38)	[9, 28, 36-38]
	Lipid peroxidation inhibitor	Ferrostatins (e.g., ferrostatin-1, SRS11-92, SRS12-45, SRS13-35, SRS13-37, and SRS16-86), liproxstatins (e.g., liproxstatin-1)	[21, 25]
	Lysosome and autophagy inhibitor	Ammonium chloride, bafilomycin A_1, chloroquine, wortmannin, 3-methyladenine, cryptotanshinone, S3I-201, CA-074Me, NH_4Cl, pepstatin A, E64	[39-42]
	Mitochondrial respiratory chain complex inhibitor	Rotenone, diethyl butylmalonate, antimycin, NaN_3	[43]
	Mitochondrial ROS inhibitor	XJB-5-131, JP4-039, mitoTEMPO, MitoQ	[44, 45]
	Neurotransmitter	Dopamine	[46]
	NOX inhibitor	Diphenyleneiodonium chloride (DPI), GKT137831, GKT136901, apocynin	[21]
	RNS inhibitor	DT-PTZ-C, 1400W	[47, 48]

Table S2. Main proteins modulating ferroptosis

Promoters	Pathway	Symbol	Description	Function	Refs
	**Calcium pathway**	ORAI1	ORAI calcium release-activated calcium modulator 1	Increase calcium influx	[31]
	**Cysteine metabolism**	ATF3	Activating transcription factor 3	Inhibit SLC7A11 expression	[49]
		BAP1	BRCA1-associated protein 1	Inhibit SLC7A11 expression	[50]
		CDO1	Cysteine dioxygenase type 1	Catalyze the conversion of L-cysteine to cysteine sulfinic acid	[51]
		TP53	Tumor protein P53	Inhibit SLC7A11 expression	[52]
	**DNA damage pathway**	ATM	ATM serine/threonine kinase	Promote radiotherapy-mediated SLC7A11 downregulation	[53]
	**Epithelial–mesenchymal transition pathway**	ZEB1	Zinc finger E-box–binding homeobox 1	Promote epithelial-to-mesenchymal transition	[54]
	**ER stress**	DDIT3/CHOP	DNA damage-inducible transcript 3	Increase ER stress	[55]
	**Glutamine metabolism**	GLS2	Glutaminase 2	Catalyze the hydrolysis of glutamine to glutamate	[34]
		GOT1	Glutamic-oxaloacetic transaminase 1	Increase αKG production	[34]
		SLC38A1	Solute carrier family 38 member 1	Increase L-glutamine uptake	[34]
	**GSH metabolism**	ABCC1/MRP1	ATP-binding cassette subfamily C member 1	Increase GSH release	[56]
	**Iron metabolism**	HMOX1	Heme oxygenase 1	Mediate heme catabolism	[14]
		IREB2	Iron-responsive element-binding protein 2	Increase iron metabolism gene expression	[21]
		TF	Transferrin	Increase iron uptake	[57]
		TFRC	Transferrin receptor	Promote iron uptake	[9]
		LTF	Lactotransferrin	Increase iron uptake	[58]
		YAP1	Yes-associated protein 1	Increase TFRC expression	[59]
	**KRAS pathway**	BRAF	B-Raf proto-oncogene, serine/threonine kinase	Activate MAPK pathway	[28]
		KRAS	KRAS proto-oncogene, GTPase	Activate MAPK pathway	[28]
	**Lipid metabolism**	ACSL4	Acyl-CoA synthetase long-chain family member 4	Increase lipid synthesis	[23, 24, 60]
		ALOX12	Arachidonate 12-lipoxygenase, 12S type	Increase lipid peroxidation	[61]
		ALOX15B	Arachidonate 15-lipoxygenase type B	Increase lipid peroxidation	[62]
		ALOXE3	Arachidonate lipoxygenase 3	Increase lipid peroxidation	[62]
		CS	Citrate synthase	Increase mitochondrial fatty acid metabolism	[21]

		CSF2	Colony-stimulating factor 2	Increase mitochondrial fatty acid metabolism	21
		EPAS1/HIF2α	Endothelial PAS domain protein 1	Increase PUFA production	63
		HILPDA	Hypoxia-inducible lipid droplet-associated	Increase PUFA production	63
		LPCAT3	Lysophosphatidylcholine acyltransferase 3	Increase lipid synthesis	24
		MDM2	MDM2 proto-oncogene	Increase PPARA activity	64
		MDMX	MDM4 regulator of P53	Increase PPARA activity	64
		PEBP1	Phosphatidylethanolamine-binding protein 1	Bind and increase ALOX15 activity	65
		POR	Cytochrome P450 oxidoreductase	Increase phospholipid peroxidation	66
		SAT1	Spermidine/spermine N1-acetyltransferase 1	Increase ALOX15 expression	67
		SQLE	Squalene epoxidase	Catalyze the oxidation of squalene to 2,3-oxidosqualene	68
		YAP1	Yes-associated protein 1	Increase ACSL4 expression	59
	**Lysosome and autophagy**	AMPK	AMP-activated protein kinase	Promote BECN1 phosphorylation and autophagosome formation	69
		ATG3	Autophagy-related 3	Promote autophagosome formation	40
		ATG5	Autophagy-related 5	Promote autophagosome formation	70
		ATG7	Autophagy-related 7	Promote autophagosome formation	70
		ATG13	Autophagy-related 13	Promote autophagosome formation	40
		ATG16L1	Autophagy-related 16-like 1	Promote autophagosome formation	71
		BECN1/ATG6	Beclin 1	Promote autophagosome formation and inhibit SLC7A11 activity	69
		ELAVL1	ELAV-like RNA-binding protein 1	Increase BECN1 expression	72
		HSP90	Heat shock protein 90	Mediate chaperone-mediated autophagy	42
		MAP1LC3B/ATG8	Microtubule-associated protein 1 light chain 3 beta	Promote autophagosome formation	73
		NCOA4	Nuclear receptor coactivator 4	Mediate ferritinophagy	70
		PINK1	PTEN-induced kinase 1	Mediate mitophagy	4
		RAB7A	RAB7A, member RAS oncogene family	Mediate lipophagy	74
		SQSTM1/p62	Sequestosome 1	Mediate clockophagy	12
		STAT3	Signal transducer and activator of transcription 3	Mediate lysosomal cell death	41
		CTSB	Cathepsin B	Promote DNA damage-induced autophagy	75
		STING1/TMEM173	Stimulator of interferon response CGAMP interactor 1	Mediate DNA damage-induced autophagy	5

		ULK1	Unc-51–like autophagy-activating kinase 1	Promote autophagosome formation	40
		ULK2	Unc-51–like autophagy-activating kinase 2	Promote autophagosome formation	40
	**Mitochondrial function**	BBC3/PUMA	BCL2-binding component 3	Increase mitochondrial membrane permeabilization	55
		BID	BH3-interacting domain death agonist	Increase mitochondrial membrane permeabilization	76
		VDAC2	Voltage-dependent anion channel 2	Increase mitochondrial membrane potential	28
		VDAC3	Voltage-dependent anion channel 3	Increase mitochondrial membrane potential	28
	**NFE2L2 pathway**	ACVR1B/ALK4	Activin A receptor type 1B	Inhibit NFE2L2 activation	77
		CDKN2A/ARF	Cyclin-dependent kinase inhibitor 2A	Inhibit NFE2L2 activation	78
		EIF2AK2/PKR	Eukaryotic translation initiation factor 2 alpha kinase 2	Inhibit NFE2L2 activation	79
		KEAP1	Kelch-like ECH-associated protein 1	Inhibit NFE2L2 activation	17
		TGFBR1/ALK5	Transforming growth factor beta receptor 1	Inhibit NFE2L2 activation	77
	**NOX pathway**	CYBB/NOX2	Cytochrome B-245 beta chain	Mediate ROS production	80
		DPP4	Dipeptidyl peptidase 4	Bind and increase NOX1 activity	33
		HDDC3/MESH1	HD domain containing 3	Mediate ROS production	81
		NOX1	NADPH oxidase 1	Mediate ROS production	33
		NOX4	NADPH oxidase 4	Mediate ROS production	82
	**Transsulfuration pathway**	CARS1	Cysteinyl-tRNA synthetase 1	Inhibit the transsulfuration pathway	83
**Repressors**	**AKR1C pathway**	AKR1C1	Aldo-keto reductase family 1 member C1	Catalyze NADPH-dependent reductions	1
		AKR1C2	Aldo-keto reductase family 1 member C2	Catalyze NADPH-dependent reductions	1
		AKR1C3	Aldo-keto reductase family 1 member C3	Catalyze NADPH-dependent reductions	1
	**BH_4 metabolism**	GCH1	GTP cyclohydrolase 1	Increase BH_4 production	84
	**Cell adhesion**	CDH1/E-cadherin	Cadherin 1	Increase cell contact	59
		ITGA6	Integrin subunit alpha 6	Increase cell adhesion	85
		ITGB4	Integrin subunit beta 4	Increase cell adhesion	85
		NECTIN4 /PVRL4	Nectin cell adhesion molecule 4	Promote cell clustering	86
		NF2/Merlin	Neurofibromin 2	Increase cell contact	59
	**CoQ10 metabolism**	AIFM2/FSP	Apoptosis-inducing factor mitochondria-associated 2	Increase CoQ10 production	7, 87
		COQ2	Coenzyme Q2, polyprenyltransferase	Increase CoQ10 production	7
	**Cysteine metabolism**	CD44/CD44v	CD44 molecule (Indian blood group)	Bind and increase SLC7A11 stabilization	88
		H2Bub1	Monoubiquitinated H2B	Increase SLC7A11 expression	89

		MUC1	Mucin 1, cell surface-associated	Bind and increase SLC7A11 activity	[90]
		OTUB1	OTU deubiquitinase, ubiquitin aldehyde-binding 1	Bind and increase SLC7A11 stabilization	[88]
		SLC3A2	Solute carrier family 3 member 2	Import cystine	[91]
		SLC7A11	Solute carrier family 7 member 11	Import cystine	[21].
	**DNA damage pathway**	FANCD2	FA complementation group D2	Inhibit DNA damage	[92]
		TFAM	Transcription factor A, mitochondrial	Protect mitochodniral DNA stress	[5]
	**ER stress**	ATF4	Activating transcription factor 4	Inhibit HSPA5 expression	[93]
		HSPA5/GRP78	Heat shock protein family A (Hsp70) member 5	Inhibit GPX4 degradation	[93]
	**GSH metabolism**	GCLC	Glutamate-cysteine ligase catalytic subunit	Mediate GSH synthesis	[34]
	**Iron metabolism**	CISD1	CDGSH iron sulfur domain 1	Inhibit mitochondrial iron uptake	[35]
		CISD2	CDGSH iron sulfur domain 2	Inhibit mitochondrial iron uptake	[94]
		FTH1	Ferritin heavy chain 1	Store iron	[17]
		FTMT	Ferritin mitochondrial	Store mitochondrial iron	[95]
		HSPB1	Heat shock protein family B (small) member 1	Inhibit iron uptake	[37]
		ISCU	Iron-sulfur cluster assembly enzyme	Increase iron-sulfur cluster assembly	[96]
		PROM2	Prominin 2	Promote iron export	[97]
		SLC40A1/FPN	Solute carrier family 40 member 1	Promote iron release	[57]
	**Lipid metabolism**	ACSL3	Acyl-CoA synthetase long-chain family member 3	Increase MUFA-CoA production	[98]
		FDFT1	Farnesyl-diphosphate farnesyltransferase 1	Increase squalene accumulation	[68]
		GPX4	Glutathione peroxidase 4	Inhibit lipid peroxidation	[99]
		HIF1A	Hypoxia-inducible factor 1 subunit alpha	Inhibit FABP3 and FABP7 expression	[12]
		PLIN2	Perilipin 2	Increase lipid storage	[100]
		SCD/SCD1	Stearoyl-CoA desaturase	Increase MUFA production	[19]
		TPD52	Tumor protein D52	Increase lipid storage	[74]
		VHL	Von Hippel-Lindau tumor suppressor	Increase β-oxidation and inhibit ALOX5	[101]
	**Membrane repair pathway**	ARNTL	Aryl hydrocarbon receptor nuclear translocator-like	Increase SLC7A11, GPX4, SOD1, TXN, NFE2L2, and CHMP5 expression	[102]
		CHMP5	Charged multivesicular body protein 5	Increase membrane repair	[103]
		CHMP6	Charged multivesicular body protein 6	Increase membrane repair	[103]

	**Mitochondrial function**	NEDD4	NEDD4 E3 ubiquitin protein ligase	Promote VDAC2 and VDAC3 degradation	[104]
	**NFE2L2 pathway**	MT1G	Metallothionein 1G	Bind heavy metals	[18]
		NFE2L2/NRF2	Nuclear factor, erythroid 2-like 2	Induce antioxidant gene expression	[17]
		NQO1	NAD(P)H quinone dehydrogenase 1	Reduce quinones to hydroquinones	[17]
	**PRDX pathway**	PRDX1	Peroxiredoxin 1	Reduce peroxides	[105]
		PRDX5	Peroxiredoxin 5	Reduce peroxides	[106]
		PRDX6	Peroxiredoxin 6	Reduce peroxides	[107]
	**RNS pathway**	CAV1	Caveolin-1	Bind and increase NOS3/eNOS activity	[48]
		NOS2/iNOS	Nitric oxide synthase 2	Catalyze the formation of nitric oxide and citruline	[108]
	**Transsulfuration pathway**	CBS	Cystathionine beta-synthase	Convert homocysteine to cystathionine	[83]
	**TXN pathway**	TXN	Thioredoxin	Catalyze the reduction of disulfides in proteins	[22]

References

1. Gagliardi, M. *et al.* Aldo-keto reductases protect metastatic melanoma from ER stress-independent ferroptosis. *Cell Death Dis* **10**, 902 (2019).
2. Otsuki, Y. *et al.* Vasodilator oxyfedrine inhibits aldehyde metabolism and thereby sensitizes cancer cells to xCT-targeted therapy. *Cancer Sci* **111**, 127-136 (2020).
3. Zielke, S. *et al.* Loperamide, pimozide, and STF-62247 trigger autophagy-dependent cell death in glioblastoma cells. *Cell Death Dis* **9**, 994 (2018).
4. Basit, F. *et al.* Mitochondrial complex I inhibition triggers a mitophagy-dependent ROS increase leading to necroptosis and ferroptosis in melanoma cells. *Cell Death Dis* **8**, e2716 (2017).
5. Li, C. *et al.* Mitochondrial DNA stress triggers autophagy-dependent ferroptotic death. *Autophagy*, 1-13 (2020).
6. Shimada, K. *et al.* Global survey of cell death mechanisms reveals metabolic regulation of ferroptosis. *Nat Chem Biol* **12**, 497-503 (2016).
7. Doll, S. *et al.* FSP1 is a glutathione-independent ferroptosis suppressor. *Nature* **575**, 693-698 (2019).
8. Alborzinia, H. *et al.* Golgi stress mediates redox imbalance and ferroptosis in human cells. *Commun Biol* **1**, 210 (2018).
9. Yang, W.S. & Stockwell, B.R. Synthetic lethal screening identifies compounds activating iron-dependent, nonapoptotic cell death in oncogenic-RAS-harboring cancer cells. *Chemistry & biology* **15**, 234-245 (2008).
10. Bittker, J.A. *et al.* Screen for RAS-Selective Lethal Compounds and VDAC Ligands - Probe 2, in *Probe Reports from the NIH Molecular Libraries Program* (Bethesda (MD); 2010).
11. Yang, W.S. *et al.* Regulation of ferroptotic cancer cell death by GPX4. *Cell* **156**, 317-331 (2014).
12. Yang, M. *et al.* Clockophagy is a novel selective autophagy process favoring ferroptosis. *Sci Adv* **5**, eaaw2238 (2019).
13. Li, Z. *et al.* Carbonic anhydrase 9 confers resistance to ferroptosis/apoptosis in malignant mesothelioma under hypoxia. *Redox Biol* **26**, 101297 (2019).
14. Kwon, M.Y., Park, E., Lee, S.J. & Chung, S.W. Heme oxygenase-1 accelerates erastin-induced ferroptotic cell death. *Oncotarget* **6**, 24393-24403 (2015).
15. Mai, T.T. *et al.* Salinomycin kills cancer stem cells by sequestering iron in lysosomes. *Nat Chem* **9**, 1025-1033 (2017).
16. Hassannia, B. *et al.* Nano-targeted induction of dual ferroptotic mechanisms eradicates high-risk neuroblastoma. *J Clin Invest* **128**, 3341-3355 (2018).
17. Sun, X. *et al.* Activation of the p62-Keap1-NRF2 pathway protects against ferroptosis in hepatocellular carcinoma cells. *Hepatology* **63**, 173-184 (2016).
18. Sun, X. *et al.* Metallothionein-1G facilitates sorafenib resistance through inhibition of ferroptosis. *Hepatology* **64**, 488-500 (2016).
19. Tesfay, L. *et al.* Stearoyl-CoA Desaturase 1 Protects Ovarian Cancer Cells from Ferroptotic Cell Death. *Cancer Res* **79**, 5355-5366 (2019).
20. Dolma, S., Lessnick, S.L., Hahn, W.C. & Stockwell, B.R. Identification of genotype-selective antitumor agents using synthetic lethal chemical screening in engineered human tumor cells. *Cancer cell* **3**, 285-296 (2003).

21. Dixon, S.J. *et al.* Ferroptosis: an iron-dependent form of nonapoptotic cell death. *Cell* **149**, 1060-1072 (2012).
22. Llabani, E. *et al.* Diverse compounds from pleuromutilin lead to a thioredoxin inhibitor and inducer of ferroptosis. *Nat Chem* **11**, 521-532 (2019).
23. Kagan, V.E. *et al.* Oxidized arachidonic and adrenic PEs navigate cells to ferroptosis. *Nat Chem Biol* **13**, 81-90 (2017).
24. Doll, S. *et al.* ACSL4 dictates ferroptosis sensitivity by shaping cellular lipid composition. *Nat Chem Biol* **13**, 91-98 (2017).
25. Friedmann Angeli, J.P. *et al.* Inactivation of the ferroptosis regulator Gpx4 triggers acute renal failure in mice. *Nat Cell Biol* **16**, 1180-1191 (2014).
26. Xie, Y. *et al.* Identification of baicalein as a ferroptosis inhibitor by natural product library screening. *Biochem Biophys Res Commun* **473**, 775-780 (2016).
27. Novgorodov, S.A. *et al.* Acid sphingomyelinase promotes mitochondrial dysfunction due to glutamate-induced regulated necrosis. *J Lipid Res* **59**, 312-329 (2018).
28. Yagoda, N. *et al.* RAS-RAF-MEK-dependent oxidative cell death involving voltage-dependent anion channels. *Nature* **447**, 864-868 (2007).
29. NaveenKumar, S.K., Hemshekhar, M., Kemparaju, K. & Girish, K.S. Hemin-induced platelet activation and ferroptosis is mediated through ROS-driven proteasomal activity and inflammasome activation: Protection by Melatonin. *Biochim Biophys Acta Mol Basis Dis* **1865**, 2303-2316 (2019).
30. Li, X. *et al.* Inhibitory Effect and Mechanism of Action of Quercetin and Quercetin Diels-Alder anti-Dimer on Erastin-Induced Ferroptosis in Bone Marrow-Derived Mesenchymal Stem Cells. *Antioxidants (Basel)* **9** (2020).
31. Henke, N. *et al.* The plasma membrane channel ORAI1 mediates detrimental calcium influx caused by endogenous oxidative stress. *Cell Death Dis* **4**, e470 (2013).
32. Maher, P. *et al.* The role of Ca(2+) in cell death caused by oxidative glutamate toxicity and ferroptosis. *Cell Calcium* **70**, 47-55 (2018).
33. Xie, Y. *et al.* The Tumor Suppressor p53 Limits Ferroptosis by Blocking DPP4 Activity. *Cell Rep* **20**, 1692-1704 (2017).
34. Gao, M., Monian, P., Quadri, N., Ramasamy, R. & Jiang, X. Glutaminolysis and Transferrin Regulate Ferroptosis. *Mol Cell* **59**, 298-308 (2015).
35. Yuan, H., Li, X., Zhang, X., Kang, R. & Tang, D. CISD1 inhibits ferroptosis by protection against mitochondrial lipid peroxidation. *Biochem Biophys Res Commun* **478**, 838-844 (2016).
36. Kang, Y., Tiziani, S., Park, G., Kaul, M. & Paternostro, G. Cellular protection using Flt3 and PI3Kalpha inhibitors demonstrates multiple mechanisms of oxidative glutamate toxicity. *Nat Commun* **5**, 3672 (2014).
37. Sun, X. *et al.* HSPB1 as a novel regulator of ferroptotic cancer cell death. *Oncogene* **34**, 5617-5625 (2015).
38. Yu, Y. *et al.* The ferroptosis inducer erastin enhances sensitivity of acute myeloid leukemia cells to chemotherapeutic agents. *Mol Cell Oncol* **2**, e1054549 (2015).
39. Torii, S. *et al.* An essential role for functional lysosomes in ferroptosis of cancer cells. *Biochem J* **473**, 769-777 (2016).

40. Gao, M. *et al.* Ferroptosis is an autophagic cell death process. *Cell Res* **26**, 1021-1032 (2016).
41. Gao, H. *et al.* Ferroptosis is a lysosomal cell death process. *Biochem Biophys Res Commun* **503**, 1550-1556 (2018).
42. Wu, Z. *et al.* Chaperone-mediated autophagy is involved in the execution of ferroptosis. *Proc Natl Acad Sci U S A* **116**, 2996-3005 (2019).
43. Gao, M. *et al.* Role of Mitochondria in Ferroptosis. *Mol Cell* **73**, 354-363 e353 (2019).
44. Krainz, T. *et al.* A Mitochondrial-Targeted Nitroxide Is a Potent Inhibitor of Ferroptosis. *ACS Cent Sci* **2**, 653-659 (2016).
45. DeHart, D.N. *et al.* Opening of voltage dependent anion channels promotes reactive oxygen species generation, mitochondrial dysfunction and cell death in cancer cells. *Biochem Pharmacol* **148**, 155-162 (2018).
46. Wang, D. *et al.* Antiferroptotic activity of non-oxidative dopamine. *Biochem Biophys Res Commun* **480**, 602-607 (2016).
47. Keynes, R.G. *et al.* N(10) -carbonyl-substituted phenothiazines inhibiting lipid peroxidation and associated nitric oxide consumption powerfully protect brain tissue against oxidative stress. *Chem Biol Drug Des* **94**, 1680-1693 (2019).
48. Deng, G. *et al.* Caveolin-1 dictates ferroptosis in the execution of acute immune-mediated hepatic damage by attenuating nitrogen stress. *Free Radic Biol Med* **148**, 151-161 (2020).
49. Wang, L. *et al.* ATF3 promotes erastin-induced ferroptosis by suppressing system Xc(). *Cell Death Differ* **27**, 662-675 (2020).
50. Zhang, Y. *et al.* BAP1 links metabolic regulation of ferroptosis to tumour suppression. *Nat Cell Biol* **20**, 1181-1192 (2018).
51. Hao, S. *et al.* Cysteine Dioxygenase 1 Mediates Erastin-Induced Ferroptosis in Human Gastric Cancer Cells. *Neoplasia* **19**, 1022-1032 (2017).
52. Jiang, L. *et al.* Ferroptosis as a p53-mediated activity during tumour suppression. *Nature* **520**, 57-62 (2015).
53. Lang, X. *et al.* Radiotherapy and Immunotherapy Promote Tumoral Lipid Oxidation and Ferroptosis via Synergistic Repression of SLC7A11. *Cancer Discov* **9**, 1673-1685 (2019).
54. Viswanathan, V.S. *et al.* Dependency of a therapy-resistant state of cancer cells on a lipid peroxidase pathway. *Nature* **547**, 453-457 (2017).
55. Hong, S.H. *et al.* Molecular crosstalk between ferroptosis and apoptosis: emerging role of ER stress-induced p53-independent PUMA expression. *Oncotarget* **8**, 115164-115178 (2017).
56. Cao, J.Y. *et al.* A Genome-wide Haploid Genetic Screen Identifies Regulators of Glutathione Abundance and Ferroptosis Sensitivity. *Cell Rep* **26**, 1544-1556 e1548 (2019).
57. Ma, S., Henson, E.S., Chen, Y. & Gibson, S.B. Ferroptosis is induced following siramesine and lapatinib treatment of breast cancer cells. *Cell Death Dis* **7**, e2307 (2016).
58. Wang, Y., Liua, Y., Liua, J., Kang, R. & Tang, D. NEDD4L-Mediated LTF Protein Degradation Limits Ferroptosis. *Biochem Biophys Res Commun* (2020).

59. Wu, J. *et al.* Intercellular interaction dictates cancer cell ferroptosis via NF2-YAP signalling. *Nature* **572**, 402-406 (2019).
60. Yuan, H., Li, X., Zhang, X., Kang, R. & Tang, D. Identification of ACSL4 as a biomarker and contributor of ferroptosis. *Biochem Biophys Res Commun* **478**, 1338-1343 (2016).
61. Chu, B. *et al.* ALOX12 is required for p53-mediated tumour suppression through a distinct ferroptosis pathway. *Nat Cell Biol* **21**, 579-591 (2019).
62. Yang, W.S. *et al.* Peroxidation of polyunsaturated fatty acids by lipoxygenases drives ferroptosis. *Proc Natl Acad Sci U S A* **113**, E4966-4975 (2016).
63. Zou, Y. *et al.* A GPX4-dependent cancer cell state underlies the clear-cell morphology and confers sensitivity to ferroptosis. *Nat Commun* **10**, 1617 (2019).
64. Venkatesh, D. *et al.* MDM2 and MDMX promote ferroptosis by PPARalpha-mediated lipid remodeling. *Genes Dev* **34**, 526-543 (2020).
65. Wenzel, S.E. *et al.* PEBP1 Wardens Ferroptosis by Enabling Lipoxygenase Generation of Lipid Death Signals. *Cell* **171**, 628-641 e626 (2017).
66. Zou, Y. *et al.* Cytochrome P450 oxidoreductase contributes to phospholipid peroxidation in ferroptosis. *Nat Chem Biol* **16**, 302-309 (2020).
67. Ou, Y., Wang, S.J., Li, D., Chu, B. & Gu, W. Activation of SAT1 engages polyamine metabolism with p53-mediated ferroptotic responses. *Proc Natl Acad Sci U S A* **113**, E6806-E6812 (2016).
68. Garcia-Bermudez, J. *et al.* Squalene accumulation in cholesterol auxotrophic lymphomas prevents oxidative cell death. *Nature* **567**, 118-122 (2019).
69. Song, X. *et al.* AMPK-Mediated BECN1 Phosphorylation Promotes Ferroptosis by Directly Blocking System Xc(-) Activity. *Curr Biol* **28**, 2388-2399 e2385 (2018).
70. Hou, W. *et al.* Autophagy promotes ferroptosis by degradation of ferritin. *Autophagy* **12**, 1425-1428 (2016).
71. Zhang, Z. *et al.* RNA-binding protein ZFP36/TTP protects against ferroptosis by regulating autophagy signaling pathway in hepatic stellate cells. *Autophagy*, 1-24 (2019).
72. Zhang, Z. *et al.* Activation of ferritinophagy is required for the RNA-binding protein ELAVL1/HuR to regulate ferroptosis in hepatic stellate cells. *Autophagy* **14**, 2083-2103 (2018).
73. Park, E. & Chung, S.W. ROS-mediated autophagy increases intracellular iron levels and ferroptosis by ferritin and transferrin receptor regulation. *Cell Death Dis* **10**, 822 (2019).
74. Bai, Y. *et al.* Lipid storage and lipophagy regulates ferroptosis. *Biochem Biophys Res Commun* **508**, 997-1003 (2019).
75. Kuang, f., Liu, J., lI, C., Kang, R. & Tang, d. Cathepsin B is a mediator of organelle-specific initiation of ferroptosis *Biochem Biophys Res Commun* (2020).
76. Neitemeier, S. *et al.* BID links ferroptosis to mitochondrial cell death pathways. *Redox Biol* **12**, 558-570 (2017).
77. Fujiki, K., Inamura, H., Sugaya, T. & Matsuoka, M. Blockade of ALK4/5 signaling suppresses cadmium- and erastin-induced cell death in renal proximal tubular epithelial cells via distinct signaling mechanisms. *Cell Death Differ* **26**, 2371-2385 (2019).

78. Chen, D. *et al.* NRF2 Is a Major Target of ARF in p53-Independent Tumor Suppression. *Mol Cell* **68**, 224-232 e224 (2017).
79. Hirata, Y. *et al.* Inhibition of double-stranded RNA-dependent protein kinase prevents oxytosis and ferroptosis in mouse hippocampal HT22 cells. *Toxicology* **418**, 1-10 (2019).
80. Yang, W.H. *et al.* A TAZ-ANGPTL4-NOX2 Axis Regulates Ferroptotic Cell Death and Chemoresistance in Epithelial Ovarian Cancer. *Mol Cancer Res* **18**, 79-90 (2020).
81. Ding, C.-K.C. *et al.* MESH1 is a cytosolic NADPH phosphatase that regulates ferroptosis. *Nat Metabolism* **doi.org/10.1038/s42255-020-0181-1** (2020).
82. Poursaitidis, I. *et al.* Oncogene-Selective Sensitivity to Synchronous Cell Death following Modulation of the Amino Acid Nutrient Cystine. *Cell Rep* **18**, 2547-2556 (2017).
83. Hayano, M., Yang, W.S., Corn, C.K., Pagano, N.C. & Stockwell, B.R. Loss of cysteinyl-tRNA synthetase (CARS) induces the transsulfuration pathway and inhibits ferroptosis induced by cystine deprivation. *Cell Death Differ* **23**, 270-278 (2016).
84. Kraft, V.A.N. *et al.* GTP Cyclohydrolase 1/Tetrahydrobiopterin Counteract Ferroptosis through Lipid Remodeling. *ACS Cent Sci* **6**, 41-53 (2020).
85. Brown, C.W., Amante, J.J., Goel, H.L. & Mercurio, A.M. The alpha6beta4 integrin promotes resistance to ferroptosis. *J Cell Biol* **216**, 4287-4297 (2017).
86. Brown, C.W., Amante, J.J. & Mercurio, A.M. Cell clustering mediated by the adhesion protein PVRL4 is necessary for alpha6beta4 integrin-promoted ferroptosis resistance in matrix-detached cells. *J Biol Chem* **293**, 12741-12748 (2018).
87. Bersuker, K. *et al.* The CoQ oxidoreductase FSP1 acts parallel to GPX4 to inhibit ferroptosis. *Nature* **575**, 688-692 (2019).
88. Liu, T., Jiang, L., Tavana, O. & Gu, W. The Deubiquitylase OTUB1 Mediates Ferroptosis via Stabilization of SLC7A11. *Cancer Res* **79**, 1913-1924 (2019).
89. Wang, Y. *et al.* Epigenetic regulation of ferroptosis by H2B monoubiquitination and p53. *EMBO Rep* **20**, e47563 (2019).
90. Hasegawa, M. *et al.* Functional interactions of the cystine/glutamate antiporter, CD44v and MUC1-C oncoprotein in triple-negative breast cancer cells. *Oncotarget* **7**, 11756-11769 (2016).
91. de la Ballina, L.R. *et al.* Amino Acid Transport Associated to Cluster of Differentiation 98 Heavy Chain (CD98hc) Is at the Cross-road of Oxidative Stress and Amino Acid Availability. *J Biol Chem* **291**, 9700-9711 (2016).
92. Song, X. *et al.* FANCD2 protects against bone marrow injury from ferroptosis. *Biochem Biophys Res Commun* **480**, 443-449 (2016).
93. Zhu, S. *et al.* HSPA5 Regulates Ferroptotic Cell Death in Cancer Cells. *Cancer Res* **77**, 2064-2077 (2017).
94. Kim, E.H., Shin, D., Lee, J., Jung, A.R. & Roh, J.L. CISD2 inhibition overcomes resistance to sulfasalazine-induced ferroptotic cell death in head and neck cancer. *Cancer Lett* **432**, 180-190 (2018).
95. Wang, Y.Q. *et al.* The Protective Role of Mitochondrial Ferritin on Erastin-Induced Ferroptosis. *Front Aging Neurosci* **8**, 308 (2016).

96. Du, J. *et al.* DHA inhibits proliferation and induces ferroptosis of leukemia cells through autophagy dependent degradation of ferritin. *Free Radic Biol Med* **131**, 356-369 (2019).
97. Brown, C.W. *et al.* Prominin2 Drives Ferroptosis Resistance by Stimulating Iron Export. *Dev Cell* (2019).
98. Magtanong, L. *et al.* Exogenous Monounsaturated Fatty Acids Promote a Ferroptosis-Resistant Cell State. *Cell Chem Biol* **26**, 420-432 e429 (2019).
99. Yang, W.S. *et al.* Regulation of ferroptotic cancer cell death by GPX4. *Cell* **156**, 317-331 (2014).
100. Sun, X. *et al.* The modification of ferroptosis and abnormal lipometabolism through overexpression and knockdown of potential prognostic biomarker perilipin2 in gastric carcinoma. *Gastric Cancer* **23**, 241-259 (2020).
101. Miess, H. *et al.* The glutathione redox system is essential to prevent ferroptosis caused by impaired lipid metabolism in clear cell renal cell carcinoma. *Oncogene* **37**, 5435-5450 (2018).
102. Liu, Y., Wang, Y., Liu, J., Kang, R. & Tang, D. The circadian clock protects against ferroptosis-induced sterile inflammation. *Biochem Biophys Res Commun* **525**, 620-625 (2020).
103. Dai, E., Meng, L., Kang, R., Wang, X. & Tang, D. ESCRT-III-dependent membrane repair blocks ferroptosis. *Biochem Biophys Res Commun* **522**, 415-421 (2020).
104. Yang, Y. *et al.* Nedd4 ubiquitylates VDAC2/3 to suppress erastin-induced ferroptosis in melanoma. *Nat Commun* **11**, 433 (2020).
105. Lovatt, M. *et al.* Peroxiredoxin-1 regulates lipid peroxidation in corneal endothelial cells. *Redox Biol* **30**, 101417 (2020).
106. Qi, W. *et al.* LncRNA GABPB1-AS1 and GABPB1 regulate oxidative stress during erastin-induced ferroptosis in HepG2 hepatocellular carcinoma cells. *Sci Rep* **9**, 16185 (2019).
107. Lu, B. *et al.* Identification of PRDX6 as a regulator of ferroptosis. *Acta Pharmacol Sin* **40**, 1334-1342 (2019).
108. Kapralov, A.A. *et al.* Redox lipid reprogramming commands susceptibility of macrophages and microglia to ferroptotic death. *Nat Chem Biol* **16**, 278-290 (2020).
